# Midgut transcriptome profiling of Anoplophora glabripennis, a lignocellulose degrading cerambycid beetle

**DOI:** 10.1186/1471-2164-14-850

**Published:** 2013-12-04

**Authors:** Erin D Scully, Kelli Hoover, John E Carlson, Ming Tien, Scott M Geib

**Affiliations:** Intercollege Program in Genetics at the Huck Institutes of the Life Sciences, The Pennsylvania State University, University Park, PA 16802 USA; Department of Entomology and Center for Chemical Ecology, The Pennsylvania State University, University Park, PA 16802 USA; The Schatz Center for Tree Molecular Genetics, Department of Ecosystem Science and Management, The Pennsylvania State University, University Park, PA 16802 USA; Department of Bioenergy Science and Technology (World Class University), Chonnam National University, Buk-Gu, Gwangju 500-757 Korea; Department of Biochemistry and Molecular Biology, The Pennsylvania State University, University Park, PA 16802 USA; Tropical Crop and Commodity Protection Research Unit, USDA-ARS Pacific Basin Agricultural Research Center, Hilo, HI 96720 USA

**Keywords:** Cellulase, Carboxylesterase, Cytochrome P450, Xylanase, Comparative transcriptomics, Hemicellulose, Biofuels, Beta-glucosidase

## Abstract

**Background:**

Wood-feeding insects often work in collaboration with microbial symbionts to degrade lignin biopolymers and release glucose and other fermentable sugars from recalcitrant plant cell wall carbohydrates, including cellulose and hemicellulose. Here, we present the midgut transcriptome of larval *Anoplophora glabripennis*, a wood-boring beetle with documented lignin-, cellulose-, and hemicellulose- degrading capabilities, which provides valuable insights into how this insect overcomes challenges associated with feeding in woody tissue.

**Results:**

Transcripts from putative protein coding regions of over 9,000 insect-derived genes were identified in the *A. glabripennis* midgut transcriptome using a combination of 454 shotgun and Illumina paired-end reads. The most highly-expressed genes predicted to encode digestive-related enzymes were trypsins, carboxylesterases, β-glucosidases, and cytochrome P450s. Furthermore, 180 unigenes predicted to encode glycoside hydrolases (GHs) were identified and included several GH 5, 45, and 48 cellulases, GH 1 xylanases, and GH 1 β-glucosidases. In addition, transcripts predicted to encode enzymes involved in detoxification were detected, including a substantial number of unigenes classified as cytochrome P450s (CYP6B) and carboxylesterases, which are hypothesized to play pivotal roles in detoxifying host tree defensive chemicals and could make important contributions to *A. glabripennis’* expansive host range. While a large diversity of insect-derived transcripts predicted to encode digestive and detoxification enzymes were detected, few transcripts predicted to encode enzymes required for lignin degradation or synthesis of essential nutrients were identified, suggesting that collaboration with microbial enzymes may be required for survival in woody tissue.

**Conclusions:**

*A. glabripennis* produces a number of enzymes with putative roles in cell wall digestion, detoxification, and nutrient extraction, which likely contribute to its ability to thrive in a broad range of host trees. This system is quite different from the previously characterized termite fermentation system and provides new opportunities to discover enzymes that could be exploited for cellulosic ethanol biofuel production or the development of novel methods to control wood-boring pests.

**Electronic supplementary material:**

The online version of this article (doi:10.1186/1471-2164-14-850) contains supplementary material, which is available to authorized users.

## Background

Class Hexapoda is one of the most ancient and diverse animal lineages on the planet [1], containing organisms capable of occupying many recalcitrant niches and persisting under intense environmental conditions including extreme temperatures, periods of desiccation, and exposure to toxins [2]. Many of its members are capable of thriving on suboptimal, nutritionally-deficient substrates [3], including wood-boring beetles belonging to family Cerambycidae that feed exclusively on woody tissue. Specifically, beetles in the genus *Anoplophora* are wood-boring insects of great interest because some of its members preferentially target healthy host trees and have relatively broad host ranges [4]. For example, the Asian longhorned beetle (*Anoplophora glabripennis*) was introduced from China into the United States, Canada, and several countries in Europe and has been documented to complete development in approximately 47 deciduous tree species worldwide, including several genera commonly planted as feedstock (e.g., *Salix* and *Populus*) [5]. *Acer* spp*.* (maples) are the predominant hosts in the introduced range [6, 7]. This beetle poses a significant threat to urban streetscapes, has the potential to destroy up to 35% of the urban tree canopy in its introduced range, and has already caused millions of dollars in damage to urban landscapes [8]. Wood-borers, like *A. glabripennis*, are especially challenging to control in both their natural and invasive ranges because the larvae spend 1–2 years living deep inside their host trees [9]. Natural enemies are rare and treatment of host trees with systemic insecticides is costly and has variable efficacy against *A. glabripennis* larvae [10, 11]. The most effective method for eradication is destruction of infested and nearby host trees and implementation of strict quarantine measures to contain the infestation. Therefore, understanding the digestive physiology of this cerambycid at the genetic level is paramount to devising novel control strategies.

Because *A. glabripennis* spends the majority of its lifecycle in the larval stage and feeds primarily in the heartwood of a broad range of healthy deciduous trees, it must overcome challenges of digesting intractable woody tissue in order to acquire sufficient nutrients to complete development [7, 12]. Glucose is a predominant wood sugar, but it is present in the form of complex polysaccharides, including cellulose, hemicellulose, callose, and pectin, which are inherently difficult to digest and require a complex of hydrolytic enzymes for efficient degradation and liberation of sugar monomers [13]. Extensive hydrogen bonding coupled with linear configurations increases the crystallinity of these cell wall polysaccharides and decreases their permeability, further hindering the activity of hydrolytic enzymes. Plant cell wall polysaccharides are further protected from hydrolytic enzymes by lignin, a biopolymer containing over 12 types of chemical bonds that is extensively cross-linked to both cellulose and hemicellulose [14], shielding them from digestion. Due to the random, heterogeneous nature of these cross-linkages and the high resilience of carbon-carbon and β-aryl ether linkages that dominate this macromolecule, lignin polymers can only be efficiently degraded through oxidative depolymerization, a process that has only been conclusively documented to be catalyzed by enzymes produced by a small number of wood degrading fungi [15]. Nitrogen is also extremely limited in woody tissues [16] and plant cell wall proteins are intricately cross-linked with lignin and cellulose, making them difficult to access [17]. Other essential nutrients, including fatty acids, sterols, and vitamins are present in low concentrations or are completely absent [18]. Lastly, wood-feeding insects must overcome plant secondary metabolites that often accumulate to high concentrations in the heartwood through detoxification or sequestration processes [19].

Many wood-feeding beetles cultivate extracellular symbiotic fungi to facilitate digestion of woody tissue and nutrient acquisition, which are carried in mycangia or other specialized structures on their body [20]. For example, bark beetles utilize a mass attack strategy, in which a mycangial fungus is directly inoculated into a host tree during oviposition to facilitate pre-digestion of woody tissue and mitigation of host tree defenses. An alternative strategy is to preferentially colonize stressed trees [21] whose woody components have already been pre-digested by wood-rotting microbes. However, *A. glabripennis* is distinct from many other wood-feeding beetles in the sense that a single larvae can successfully develop in a healthy tree without requiring mass attack and the majority of the challenging reactions, including digestion of lignocellulose and hemicellulose and detoxification of plant metabolites, can occur within the gut itself [22–24]. While the midgut community associated with *A. glabripennis* has the metabolic potential to overcome many of the challenges associated with feeding in woody tissue, including degradation of lignin, cellulose, and hemicellulose and acquisition of nitrogen and other essential nutrients [25], the contributions of insect-derived digestive and nutrient acquiring enzymes cannot be ignored since insects themselves can produce a diverse array of digestive enzymes, including cellulases, hemicellulases, pectinases, and enzymes that enhance lignin degradation [26–28]. Insects have also evolved other sophisticated abilities to evade host plant defenses and often possess extensive suites of enzymes involved in detoxification of plant metabolites and phytohormones, digestive proteinase inhibitors [29], and cyanates and cyanoamino acids [30], as well as enzymes capable of disrupting jasmonic acid signaling pathways [31]. Furthermore, insects produce many cytochrome P450s [32], which are integrally involved in xenobiotic metabolic processes that ultimately lead to oxidative destruction of toxic compounds, including plant derived secondary metabolites and pesticides [33].

The primary goals of this study were to survey the endogenous digestive and physiological capabilities of larval *A. glabripennis* through shotgun sequencing of midgut derived messenger RNA and to identify insect-derived genes that are highly expressed in the midgut while actively feeding in wood. The *A. glabripennis* midgut transcriptome library was also compared to all publically available transcriptome libraries sampled from other plant feeding insects to identify core groups of genes that are associated with digestive processes that could facilitate nutrient recovery from woody tissue regardless of insect taxa. This study represents an important addendum to the growing database of genomic and transcriptomic resources available for coleopterans and fills an important gap, representing the first transcriptome sampled from a wood-feeding cerambycid and the first comprehensive analysis of endogenous genes associated with wood-feeding in insects. These findings offer unique opportunities to bioprospect for enzymes that could be exploited for cellulosic biofuel production or other industrial processes, and to develop novel control methods for this destructive wood-boring pest and other wood-feeding insects.

## Results and discussion

### 454- and Illumina-Based Transcriptome Sequencing

To develop a comprehensive profile of the endogenous digestive and physiological capabilities of *A. glabripennis*, mRNA was collected from the midguts of third instar larvae feeding in the heartwood of a preferred host (*Acer saccharum*) and was sequenced using both Roche 454 pyrosequencing and Illumina technologies. In total, 232,824 shotgun sequence reads were produced using the Roche 454 FLX platform using two separate runs. 173,778 reads (35.7 Mb), ranging in length from 26 to 557 nt (average read length: 205 nt), were generated on a half plate and 59,046 reads (13.5 Mb) ranging from 39 to 407 nt (average read length: 228 nt), were generated on a quarter plate. These runs correspond to E4GEBH102.sff and E5TY7PB02.sff from SRA [SRX265389], respectively. Reads from both runs were pooled and were quality filtered and assembled together. Approximately 210,000 (42 Mb) of the total 454 FLX reads passed quality filtering and were utilized in the assembly. To enhance sequencing depth and acquire a more complete inventory of the endogenous digestive and metabolic capabilities of *A. glabripennis*, 130 million 100 bp paired Illumina reads (36 Gb) with a library insert size of 175 nucleotides (nt) were generated on a single lane using the Illumina HiSeq 2000 (SRX265394). After quality filtering and adapter removal, over 128 million read pairs (34 Gb) remained and were utilized in downstream processing and analyses. Digital k-mer normalization reduced the number of Illumina read pairs to 2,090,296, which were ultimately used for co-assembly with the 454 FLX reads.

### Assembly and Annotation Statistics

#### ***454 Assembly and Annotation Statistics for Comparative Transcriptomics***

**Figure 1:**
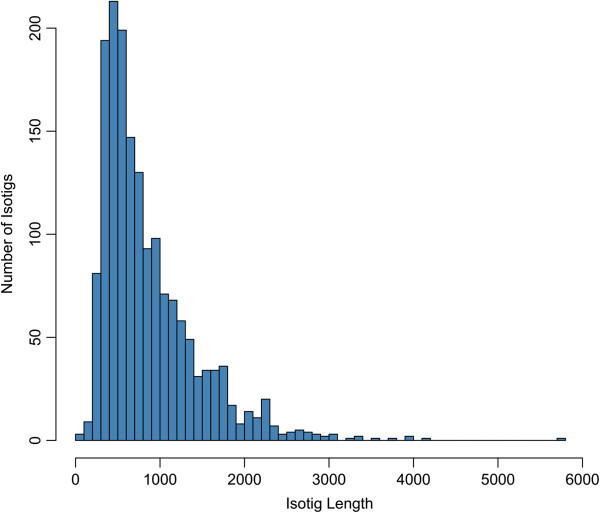
**Histogram of isotig lengths generated from 454 FLX reads.** 232,824 shotgun reads were assembled into 1,658 isotigs (1.4 Mb) using Newbler, which represent assembled transcripts. The N50 isotig length was 1,076 nt.

To facilitate comparisons to transcriptome libraries prepared from the guts of other herbivorous insects, which were derived solely from 454 reads, the 454 reads were first assembled and analyzed without the Illumina reads. Of the 232,824 shotgun reads generated through 454 pyrosequencing (49.2 Mb), approximately 191,000 reads assembled into 2,081 contigs (1.26 Mb), ranging in length from 200 nt to 5,701 nt with an N50 contig length of 907 nt (Figure 1). Assembled contigs that shared common reads were placed into isogroups. These contigs are often broken at branch points between exon boundaries in multiple transcript isoforms from the same unigene. Contig branch structures within each isogroup were then traversed to create 1,658 isotigs (1.4 Mb), which represent unique assembled transcripts or transcript fragments. The N50 isotig length was 1,076 nt and isotigs were grouped into 1,475 isogroups, representing a gene locus or unigene. Of these isogroups, 1,360 were comprised of a single transcript isoform and the average number of isotigs within an isogroup was 1.1. The maximum number of isotigs classified to the same isogroup was 11. For downstream comparative analyses, isogroups were treated as unigenes and isotigs associated with the same isogroup were treated as transcript isoforms. Roughly 27,000 reads (4.0 Mb) were singletons and were not included in the assembly. Of the singletons, approximately 19,000 reads (3.7 Mb) were flagged as high quality and, to increase the amount of information present in the transcriptome dataset, these singleton reads were concatenated to the assembly and the pooled dataset was utilized in downstream transcriptome comparisons. Assembly metrics from the 454-based assembly are presented in Table 1. After clustering the isotigs and high quality singletons with CD-HIT-EST using a sequence similarity threshold of 0.97 to group transcripts that likely represented allelic variants of the same gene, the total number of isotigs and singletons was reduced to around 18,000. Seventy-eight of these isotigs and reads were classified as ribosomal RNAs, while none were classified as tRNAs. Roughly 10,000 isotigs and singletons had BLASTX alignments to protein sequences housed in the non-redundant protein database at an e-value threshold of 1e^-5^ or lower. Of the isotigs and singletons that had BLASTX alignments, 9,130 were classified to class Hexapoda (91%). Annotation statistics for this assembly are summarized in Table 2.

**Table 1:** **Assembly metrics for** ***A. glabripennis*** **midgut transcriptome assembly generated using 454 reads only**

Metric	Contig	Isotig
Min length (nt)	200	204
N80 length (nt)	510	592
N50 length (nt)	907	1076
N20 length (nt)	1577	1753
Max length (nt)	5701	5701

**Table 2:** **Annotation statistics for** ***A. glabripennis*** **midgut transcriptome 454 assembly**

Number of Unique Reads and Isotigs	20,587
Number of Reads and Isotigs with BLASTX Hits	10,030
Number of Insect Reads and Isotigs	9,109
Number of Bacterial Reads and Isotigs	45
Number of Fungal Reads and Isotigs	30
Number of rRNAs	78
Number of tRNAs	0
Number of Insect Reads/Contigs with GO Assignments	4,331
Number of Insect Reads/Contigs with KEGG Assignments	1,633
Number of Insect Reads/Contigs with Pfam Assignments	7,202

#### ***Hybrid Illumina/454 Transcriptome Assembly***

**Figure 2:**
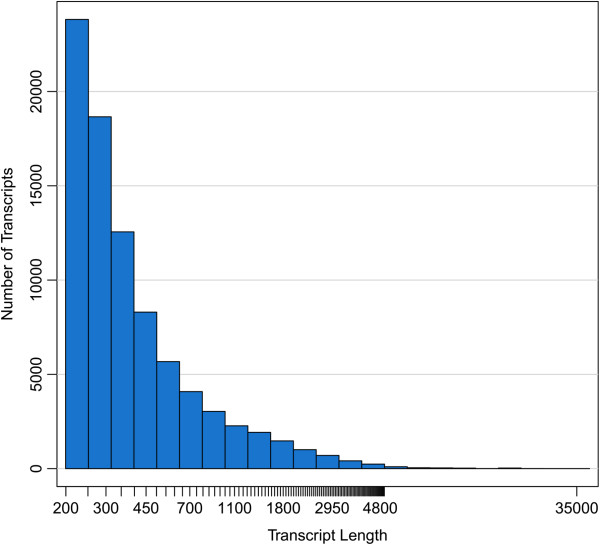
**Histogram of transcript lengths generated from 454 FLX and Illumina paired end reads using Trinity.** Co-assembly of 454 shotgun reads and Illumina paired-end sequences using Trinity yielded 42,085 transcripts (31 Mb) ranging in length from 200 to 32,701 nt with an N50 transcript length of 945 nt. This represented a substantial improvement over the 454 assembly and generated more full length transcripts in comparison.

Co-assembly with Illumina paired-end sequences using Trinity substantially improved the assembly metrics, resulting in the assembly of more full-length transcripts. For this reason, discussion of the digestive and metabolic capabilities of *A. glabripennis* are focused mainly on genes and transcripts detected in the co-assembly and the 454-only assembly is used strictly for comparisons to other herbivorous insect gut transcriptomes. The final 454/Illumina co-assembly contained 42,085 transcripts (31 Mb) ranging in length from 200 to 32,701 nt with an N50 transcript length of 945 nt (Figure 2). Approximately 14,600 transcripts had predicted protein coding regions and, of these, over 10,000 transcripts contained full length open reading frames (ORFs) with discernible start and stop codons. These transcripts were classified to 35,948 unigenes, bringing the average number of transcript isoforms per locus to 1.2. The highest number of isoforms detected for an individual gene/locus was 26 and transcripts assigned to this unigene were predicted to encode tropomyosin. Full assembly and annotation metrics for the 454-Illumina hybrid assembly are presented in Table 3. Of the unigenes predicted to contain full length or partial ORFs, 13,892 (99%) had BLASTP alignments at an e-value threshold of 1e^-5^ or lower, while 341 unigenes were predicted to encode rRNAs and 70 transcripts were predicted to encode tRNAs. Approximately 9,900 (72%) of the unigenes that had BLASTP alignments were classified to class Hexapoda. Annotation metrics are presented in Table 4. To assess the potential completeness and quality of the larval midgut transcriptome assembly, several KEGG metabolic pathways known to be conserved, functional, and complete in insects were examined to determine if all genes associated with these pathways were represented in the assembly. Full pathways for glycolysis and gluconeogenesis, pyrimidine metabolism, purine metabolism, pyruvate metabolism, the citric acid cycle, and phosphatidylinositol signaling systems were successfully constructed from protein coding transcripts in the assembly.

**Table 3:** **Assembly metrics for** ***A. glabripennis*** **midgut transcriptome Illumina-454 co-assembly**

Number of transcripts	Min transcript length (nt)	N80 transcript length (nt)	N50 transcript length (nt)	N20 transcript length (nt)	Max transcript length (nt)
42,085	200	439	945	2407	32,701

**Table 4:** **Annotation statistics for** ***A. glabripennis*** **midgut transcriptome Illumina-454 hybrid assembly**

Number of Unigenes	42,085
Number of Unigenes with BLASTP Hits	13,892
Number of Insect Unigenes	9,959
Number of rRNAs	341
Number of tRNAs	70
Number of Insect Unigenes with GO Assignments	5,066
Number of Insect Unigenes with KEGG Assignments	1,685
Number of Insect Unigenes with Pfam Assignments	7,688

**Figure 3:**
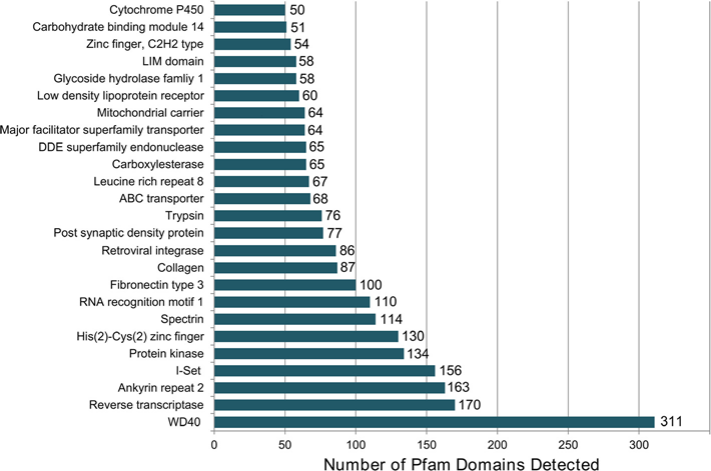
**Relative abundance of the 25 most abundant Pfam domain assignments in the** ***A. glabripennis*** **midgut transcriptome assembly.** Unigenes generated from the 454/Illumina assembly were scanned for Pfam HMMs. Over 7,500 unigenes contained Pfam domains.

**Figure 4:**
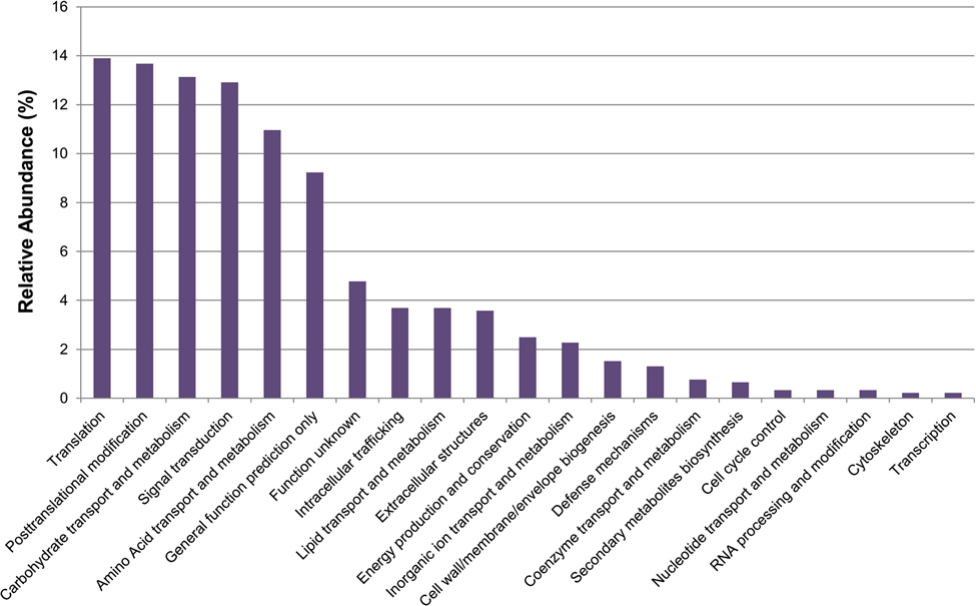
**KOG assignments for midgut unigenes.** Unigenes detected in the *A. glabripennis* midgut transcriptome were assigned to 24 different eukaryotic clusters of orthologous gene (KOG) categories. Overall, the majority of the unigenes were assigned to the signal transduction mechanisms and the general functional prediction only KOG categories.

Overall, the most abundant Pfam assignments detected in transcripts generated from the Illumina/454 co-assembly were primarily structural domains, including WD 40, ankyrin, spectrin, and I-set, and domains associated with regulatory proteins, including reverse transcriptase, protein kinases, and zinc finger domain proteins. The most dominant unigenes predicted to encode enzymes that were detected in this assembly were annotated as trypsins, DDE superfamily endonucleases, carboxylesterases, cytochrome P450s, and glycoside hydrolase family one (Figure 3). The majority of the unigenes detected in the midgut were assigned to the general functional prediction KOG category, indicating that many of the unigenes detected in the midgut have not been definitively assigned to metabolic pathways and suggesting that they may be involved in novel or uncharacterized processes. Other highly abundant KOG categories included signal transduction and carbohydrate transport and metabolism (Figure 4). KOG assignments of unigenes with putative signal peptides that could be involved in digestive processes were also conducted (Additional file 1: Figure S1).

### Glycoside Hydrolases and Plant Cell Wall Digesting Enzymes

#### ***Transcripts Predicted to Encode Hemicellulases***

**Figure 5:**
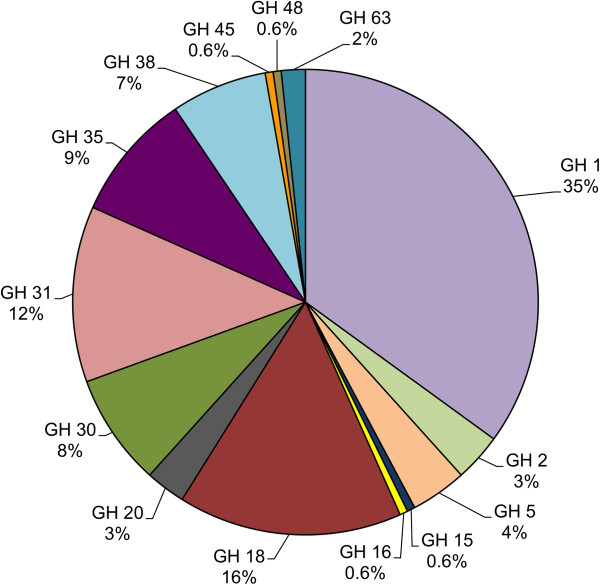
**Distribution of glycoside hydrolase families found in the** ***A. glabripennis*** **midgut transcriptome.** Approximately 180 unique unigenes assigned to 14 different glycoside hydrolase families were detected. The most dominant families were GH 1 and 18 while GH families 15, 16, 45, and 48 were represented by a single unigene.

Over 180 different unigenes assigned to 14 GH families were identified, many of which have annotations consistent with involvement in plant cell wall degradation in the *A. glabripennis* midgut (Figure 5). Of particular interest are enzymes capable of degrading cellulose and hemicellulose, which are the two most predominant polysaccharides found in hardwoods. Few insect enzymes involved in large-scale degradation of xylan (the dominant form of hemicellulose found in most deciduous trees) [34] have been expressed and biochemically characterized *in vitro*. Through in-gel zymograms infused with birch xylan and MADLI-TOF-based peptide sequencing, it was previously demonstrated that *A. glabripennis* was capable of producing at least one enzyme with hydrolytic activity directed at birch xylan, suggesting that the beetle has the endogenous capacity to degrade this hardwood polysaccharide [24]. Eight transcript isoforms of this GH 1 xylanase were detected in the transcriptome assembly, indicating that xylan degrading transcripts in *A. glabripennis* may be more numerous that previously reported. The identification of these transcripts is significant and redefines the role of insects in processing xylan as it has generally been presumed that xylanases are only produced by microbial symbionts [35]. It is possible that other GH transcripts detected in the *A. glabripennis* midgut may also encode xylanases or β-xylosidases. For example, GH family 30 is predominately comprised of β-xylosidases [36] and over 10 unigenes with GH 30 functional domains were detected in the *A. glabripennis* midgut transcriptome. However, the ability to predict polysaccharide substrates and catalytic potentials of these enzymes was impeded by the lack of specific annotations in the databases because very few of the highest scoring BLASTP alignments have corresponding KEGG E.C. annotations. More refined annotations would require in-depth functional genomics approaches.

Despite *A. glabripennis’* endogenous ability to degrade long-chain xylan into shorter oligosaccharides, no insect-derived transcripts capable of releasing xylose monomers (β-xylosidases) from xylo-oligomers or converting xylose to ATP or acetyl coA (volatile fatty acids) were detected. Endogenous xylose utilization capabilities have not been described in cerambycids [37] and it is generally hypothesized that these beetles depend on yeasts or other microbes in the gut to supply these enzymes [38]. Previous metagenomic profiling of the *A. glabripennis* midgut microbiota revealed that yeasts and lactic acid bacteria associated with the gut have the metabolic potential to ferment five-carbon sugars, converting them to ethanol and other compounds that could be used directly by *A. glabripennis* for energy and fatty acid production [25]. Furthermore, the presence of a large number of *A. glabripennis*-derived transcripts predicted to encode alcohol and aldehyde dehydrogenases could suggest a role in processing ethanol, acetate, and other metabolites generated through xylose fermentation by microbes colonizing the gut. *A. glabripennis* also possesses full fatty acid biosynthetic pathways capable of incorporating acetate, acetyl coA, and microbial fermentation products into fatty acids.

Other minor polysaccharides present in heartwood hemicellulose include glucuronoxylan, arabinoxylan, glucomannan, and xyloglucan, which are comprised of mannose, galactose, rhamnose, arabinose, glucuronic acid, and galacturonic acid residues residues [39]. Despite the fact that these polysaccharides make up a relatively minor component of plant cell walls in the heartwood of deciduous trees, many transcripts predicted to encode enzymes that release mannose and galactose residues from polysaccharides were detected in the *A. glabripennis* midgut. For example, 16 unigenes predicted to encode GH 35 exo-β-galactosidases and β-galactosidases, 12 unigenes predicted to encode GH 38 α-mannosidases and mannosyl-oligosaccharide α-1,3-1,6 mannosidases, and 3 unigenes predicted to encode GH 47 α-mannosidases were detected and could be utilized to liberate mannose and galactose from the hemicellulose matrix. Other transcripts predicted to encode enzymes responsible for processing minor polysaccharides present in hemicellulose included β-mannosidase, lactase, β-thioglucosidase, and β-fucosidase [36]. Further, *A. glabripennis* actively expressed transcripts involved in processing and utilizing mannose and galactose sugars via glycolysis, suggesting that these sugars can be directly utilized for energy production.

#### ***Transcripts predicted to encode cellulases and callases***

Like many other wood-feeding insects, *A. glabripennis* also produces a number of transcripts predicted to encode cellulases. One of the most striking discoveries in the midgut transcriptome was the presence of six GH 5 cellulase unigenes, which all had highest scoring BLASTP alignments to GH 5 endo-β-1,4 glucanases previously detected in the guts of other wood-feeding cerambycid beetles. Recombinant protein expression assays revealed that cellulases associated with other cerambycids (e.g., *Apriona germari*, *Oncideres albomarginata chamela*, and *Psacothea hilaris*) catalyzed the release of cello-oligomers from crystalline cellulose [26, 27, 40]. These were not flagged as transcript isoforms by Trinity, suggesting that genes encoding cellulases are represented in multiple copies in the *A. glabripennis* genome. The purpose of this redundancy is unknown, but several other coleopterans harbor multiple copies of cellulases belonging to the same GH family [41, 42]. These enzymes could function under different physiological conditions, which is consistent with the contrasting pH and oxygen gradients that can be found in different regions of cerambycid midguts [43]. Alternatively, these unigenes could encode enzymes with slightly different catalytic capabilities that act on different cellulose macromolecule substructures [44], target soluble or insoluble fractions of cellulose [45], or process cello-oligomers into cellobiose (exoglucanase activity). In addition to GH 5 cellulases, *A. glabripennis* also expresses endo-β-1,4-glucanases classified to GH families 45 and 48. *A. glabripennis* also produced a large number of β-glucosidases, which hydrolyze cellobiose to release glucose. The majority of these were classified to GH family 1, which was the most abundant GH family detected in the *A. glabripennis* midgut transcriptome. The overabundance of β-glucosidases relative to cellulases is common in many wood-feeding insects and wood-degrading microorganisms and is hypothesized to serve as a mechanism to indirectly enhance cellulase activity. These β-glucosidases can often act quickly and efficiently to release glucose from cellobiose, reducing the impact of end product inhibition on cellulase activity [46].

In addition to transcripts encoding enzymes predicted to disrupt major hardwood polysaccharides, several transcripts involved in degrading minor polysaccharides were detected. For example, callose is a linear polysaccharide comprised of β-1,3 and β-1,6 linked glucose. Although callose is normally associated with the fleshy and metabolically active regions of plants, such as leaves and stems, it is also sporadically deposited in cell walls of secondary growth [47] and represents suitable stores of glucose that could be liberated and assimilated by *A. glabripennis*. Several β-1,3 and β-1,6 glucanases detected in the midgut transcriptome could be involved in liberating glucose from this polysaccharide.

#### ***Transcripts predicted to encode enzymes that contribute to lignin and phenylpropanoid degradation***

While lignin is highly abundant in the heartwood of deciduous trees where the *A. glabripennis* larvae were collected for this study, no transcripts predicted to encode enzymes that are capable of yielding the types of lignin degradation products previously observed in *A. glabripennis* frass [22] were detected. A single laccase unigene with a signal peptide for extracellular targeting was detected in addition to several extracellular copper oxidase domain proteins, peroxidases, aldo-keto reductases, and alcohol dehydrogenases. Laccases are involved in lignin degradation in some white rot fungal taxa [48], and an endogenous termite laccase capable of degrading lignin alkali and lignin phenolics was recently characterized [49]. However, despite their reported ability to degrade lignin phenolics, many laccases require extracellular redox mediators to disrupt the non-phenolic β-aryl ether and C-C linkages that dominant hardwood lignins to yield the types of degradation products observed in *A. glabripennis* frass [50]. While pathways for synthesis of these redox mediators have been identified in some white rot fungi, insects are unlikely to have the endogenous ability to synthesize them since all characterized laccase redox mediators are comprised of aromatic rings, which insects cannot inherently synthesize [51]. Based on these observations, we hypothesize that lignin degrading activities in the gut should be directly enhanced through interactions with microbial enzymes capable of synthesizing aromatic redox mediators or liberating aromatic compounds from lignin. Lignin metabolites released from the biopolymer can also be used as laccase mediators. In addition to laccases, 26 unigenes predicted to encode aldo-keto reductases were detected in the *A. glabripennis* transcriptome. In a recent study, expression levels of termite-produced aldo-keto reductases were correlated with feeding on wood and a recombinant aldo-keto reductase expressed in conjunction with other termite-derived cellulases enhanced sugar release from pine saw dust [52], suggesting a role in enhancing lignocellulose digestion. Additionally, aldo-keto reductases have been shown to enhance xylose metabolism [53], degrade xenobiotics and carbohydrates [54], function as aryl alcohol dehydrogenases to facilitate the degradation of β-aryl ethers in lignin [55], and are induced by exposure to phenolics and aromatic compounds in bacteria and yeasts [56]. The abundance of these aldo-keto reductases in the midgut suggests that they could work in collaboration with other insect and microbial enzymes to facilitate penetration of lignin.

Other enzymes encoded by the *A. glabripennis* transcriptome capable of disrupting bonds that cross-link hemicellulose to lignin [57] included esterases, which liberate polysaccharide termini from the cell wall matrix, exposing them to hydrolytic enzymes and enhancing sugar release from this group of polysaccharides. Additionally, 16 unigenes predicted to encode alcohol dehydrogenases were detected in the midgut transcriptome; although these enzymes have not been shown to break linkages in polymeric lignin, they are hypothesized to enhance lignin oxidation in the guts of termites [58] and they could serve similar roles in the *A. glabripennis* midgut. Finally, a number of extracellular peroxidases were also detected. Although the roles of insect-derived peroxidases in digestion and physiology are numerous and diverse [59], direct roles for insect peroxidases in lignin degradation have not been explored.

Lignin degradation releases phenylpropanoids (e.g. coumaryl and cinnamyl alcohol), which are often toxic; however, *A. glabripennis* produces enzymes capable of degrading phenylpropanoid subunits, including epoxide hydrolases, which are often involved in polycyclic aromatic compound metabolism [60]. Other transcripts predicted to encode detoxification enzymes and antioxidants that could make contributions to degradation or inactivation of toxic lignin metabolites include alcohol dehydrogenases, aldehyde dehydrogenases, cytochrome P450s, glutathione S-transferases, catalases, carboxylesterases, enzymes involved in aromatic compound degradation, and glucuronosyl transferases. Additionally, aldo-keto reductases are capable of degrading phenolic compounds, including tannins and phenylpropanoids released from lignin degradation, and could be primed for detoxification roles.

### Transcripts predicted to encode detoxification enzymes

*A. glabripennis* eggs hatch directly beneath the bark of hardwood trees and first and second instars feed on primary phloem and xylem [6], which serve as diffuse transport systems for toxic tree defensive compounds [61], before tunneling into the heartwood as later instars. Though heartwood is not as metabolically active as the primary phloem and xylem, it accumulates potentially toxic secondary metabolites, including alkaloids, tannins, hydroxycinnamic acids, and phenolic glycosides, defending the plant from herbivory and protecting structural polysaccharides and biopolymers from biotic assaults [62]. Given that *A. glabripennis* completes development in over 47 different tree species [9] and that it feeds in the phloem and xylem before eventually making its way into the heartwood, this insect must have mechanisms to detoxify or sequester the breadth of defensive plant secondary metabolites it encounters throughout its life cycle.

The gut represents the first line of defense against ingested host plant allelochemicals, pesticides, and other toxins and many transcripts predicted to encode detoxification enzymes were detected. For example, 50 cytochrome P450-like unigenes were detected in the *A. glabripennis* midgut transcriptome. These enzymes have versatile oxidoreductive properties, are highly involved in degrading lipophilic toxins [63, 64], and have been shown to confer resistance to pesticides [32] as well as small aromatic toxins that can accumulate to high concentrations in the heartwood of trees (e.g. alkaloids). The majority of the cytochrome P450 unigenes detected in the *A. glabripennis* midgut transcriptome had highest scoring BLASTP alignments to cytochrome P450s identified in the *Tribolium castaneum* genome [65, 66], although the percent similarity at the amino acid level ranged from 34% to 66%, reflecting a relatively large degree of divergence from previously annotated cytochrome P450s (Table 5).

**Table 5:** **Cytochrome P450 annotations from** ***A. glabripennis*** **midgut transcriptome**

Protein ID	Length (Amino Acid)	Highest Scoring BLAST alignment (Accession Number)	Organism	% Amino Acid Identity	Clan	Putative cyp450 family assignment	Full or partial CDS
m.43	203	EFA02818	*T. castaneum*	55%	3	CYP6B	5′ Partial
m.576	221	EFA07581	*T. castaneum*	54%	Mito	CYP12H	5′ Partial
m.2790	273	AAZ94271	*L. decemlineata*	53%	3	CYPB6	3′ Partial
m.2949	135	EFA12856	*T. castaneum*	53%	3	CYP345	Internal Partial
m.4110	203	EFA05693	*T. castaneum*	54%	3	CYP6B	3′ Partial
m.4694	288	EEZ97722	*T. castaneum*	62%	Mito	CYP314	Internal Partial
m.4920	134	EFA04616	*T. castaneum*	60%	4	CYP4B	Internal Partial
m.5185	243	EFA02818	*T. castaneum*	44%	3	CYP6B	3′ Partial
m.5186	327	ADH29767	*T. castaneum*	43%	3	CYP6B	Internal Partial
m.5187	430	ADH29767	*T. castaneum*	44%	3	CYP6B	5′ Partial
m.5554	462	EFA10756	*T. castaneum*	59%	4	CYP4Q	Complete
m.5960	494	NP_001034529	*T. castaneum*	55%	4	CYP4Q	Complete
m.5973	415	XP_001809620	*T. castaneum*	41%	4	CYP347	Complete
m.6105	269	EFA05693	*T. castaneum*	52%	3	CYP6B	3′ Partial
m.6106	194	EFA12631	*T. castaneum*	67%	3	CYP6B	5′ Partial
m.6149	509	EFA12857	*T. castaneum*	44%	3	CYP345	5′ Partial
m.6332	450	XP_972348	*T. castaneum*	55%	3	CYP9	Complete
m.6566	194	EFA02818	*T. castaneum*	47%	3	CYP6B	Complete
m.6961	509	EFA12636	*T. castaneum*	58%	3	CYP6B	Complete
m.7294	504	EFA12856	*T. castaneum*	54%	3	CYP345	Complete
m.7332	283	EFA12632	*T. castaneum*	55%	3	CYP6B	Internal Partial
m.7528	433	EFA04535	*T. castaneum*	48%	4	CYP4B	Internal Partial
m.7574	441	ADH29761	*T. castaneum*	53%	3	CYP6B	Complete
m.7944	199	AAP94193	*T. castaneum*	58%	4	CYP4Q	5′ Partial
m.7945	226	AAF70178	*T. castaneum*	53%	4	CYP4Q	Complete
m.8049	512	EFA02821	*T. castaneum*	54%	3	CYP6B	Complete
m.8168	513	EFA02819	*T. castaneum*	53%	3	CYP6B	Complete
m.8218	502	EFA01323	*T. castaneum*	62%	4	CYP4B	Complete
m.8553	320	EFA09242	*T. castaneum*	52%	3	CYP9Z	Complete
m.8554	369	EFA09242	*T. castaneum*	52%	3	CYP9Z	Complete
m.8555	529	EFA09242	*T. castaneum*	52%	3	CYP9Z	Complete
m.9305	514	AAZ94272	*L. decemlineata*	58%	3	CYPB6	5′ Partial
m.9306	308	AAZ94272	*L. decemlineata*	58%	3	CYPB6	3′ Partial
m.9307	224	AAZ94272	*L. decemlineata*	57%	3	CYP6B	3′ Partial
m.9308	446	AAZ94272	*L. decemlineata*	60%	3	CYP6B	Complete
m.9560	513	AAZ94272	*L. decemlineata*	60%	3	CYP6B	Complete
m.9606	332	EFA10753	*T. castaneum*	48%	4	CYP4Q	3′ Partial
m.9607	497	EFA10753	*T. castaneum*	55%	4	CYP4Z	Complete
m.9803	370	XP_9696331	*T. castaneum*	54%	3	CYP6B	Complete
m.9916	481	EFA12632	*T. castaneum*	54%	3	CYP6B	5′ Partial
m.9917	339	EFA12626	*T. castaneum*	60%	3	CYP6B	Complete
m.9918	360	AAZ94272	*L. decemlineata*	59%	3	CYP6B	Complete
m.9919	480	EFA12634	*T. castaneum*	55%	3	CYP6B	5′ Partial
m.10025	360	EFA07581	*T. castaneum*	54%	Mito	CYP12H	Complete
m.10234	506	EFA12628	*T. castaneum*	52%	3	CYP6B	Complete
m.10236	390	EFA12628	*T. castaneum*	53%	3	CYP6B	5′ Partial
m.10796	118	EFA07581	*T. castaneum*	53%	Mito	CYP12H	5′ Partial
m.12025	147	AAZ94272	*L. decemlineata*	56%	3	CYP6B	3′ Partial
m.12358	270	XP_970699	*T. castaneum*	34%	N/A	N/A	5′ Partial
m.13759	245	EFA12627	*T. castaneum*	54%	3	CYP6B	Internal Partial

Cytochrome P450 unigenes detected in the *A. glabripennis* midgut were putatively assigned to clans and families based on the annotations of the highest scoring BLASTP alignments [65]. To conform to cytochrome P450 classification conventions, only alignments sharing ≥ 40% amino acid identity were used to annotate these unigenes. While a handful of unigenes predicted to encode mitochondrial cytochrome P450s were identified, non-mitochondrial clans 3 and 4 were more highly represented in comparison. Non-mitochondrial cytochrome P450s were classified to five families, which were predominated by CYP6 (27 unigenes), but also included CYP4 (8 unigenes), CYP9 (4 unigenes), CYP345 (3 unigenes), and CYP347 (1 unigene). CYP6 family genes are often present in multiple copies, occur in clusters in insect genomes [67], have pivotal roles in the detoxification of host plant defensive chemicals such as xanthotoxins, gossypol, and chlorogenic acid (a key intermediate in lignin biosynthesis) [33], and are often induced in herbivorous insects during periods of feeding.

Unigenes predicted to encode carboxylesterases were the most dominant enzyme with putative involvement in detoxification processes detected in the midgut; 65 individual unigenes were predicted to encode these proteins. Although secreted carboxylesterases are generally involved in pheromone metabolism [68], intracellular carboxylesterases are often implicated in pesticide and allelochemical metabolism and tolerance [69]. Over 30 of the carboxylesterase unigenes detected in the *A. glabripennis* midgut lacked secretory peptides and may be primed to serve detoxification roles. For example, carboxylesterases are hypothesized to mediate resistance to phenolic glycosides in *Papilio canadensis*[70], and are often found in high levels in the midgut [71]. Notably, trees in the family Salicaceae, which include many of *A. glabripennis’* preferred hosts (e.g. *Populus* spp.), are notorious producers of phenolic glycosides (e.g. salicin and tremulacin) [72] and the abundance of carboxylesterases may promote colonization and survival in these hosts.

Transcripts predicted to encode enzymes involved in conjugative deactivation of xenobiotic compounds were also detected from 30 and 21 unigenes predicted to encode UDP-glucuronosyl transferases and glutathione S-transferases, respectively [73]. These transferases can bind to xenobiotic compounds containing a diversity of functional groups, including oxygen, nitrogen, sulfur or carboxyl groups, enhancing their solubility and allowing them to be excreted or stored in the fat body for eventual elimination [74]. They have been previously shown to detoxify cyanates and cinnamaldehydes [75], which can be found in high concentrations in heartwood. Further, they can also conjugate and eliminate aromatic compounds, including tannins and toxic aromatic compounds stored in the heartwood or released from lignin degradation [76].

### Transcripts predicted to encode enzymes involved in nitrogen acquisition

Although nitrogen is scarce in the woody tissue of host trees, like other insects, *A. glabripennis* larvae have high demands for nitrogen during growth and development. Although nitrogen sources are present in very low abundance in woody tissues, microbes associated with the midgut have the metabolic capacity to synthesize all 23 amino acids, which could be assimilated and stored by the insect in the form of arylphorin and hexameric storage proteins encoded by *A. glabripennis*[25]. However, the insect possesses endogenous pathways for amino acid synthesis that may be complemented or augmented by microbial pathways, including complete pathways for the synthesis of alanine, aspartic acid, asparagine, proline, cysteine, glycine, and serine and for the synthesis of tyrosine from phenylalanine. In addition, nearly complete pathways for the synthesis of arginine, glutamic acid and selenocysteine were detected, but argininosuccinate lyase, glutamate formiminotransferase, and selenocysteine synthases transcripts were absent. These pathways may be incomplete because transcripts encoding these enzymes are simply not expressed in the midgut, they were expressed at low levels and were not detected at the sequencing depth obtained, or because they may be complemented by microbial enzymes that catalyze these reactions.

**Figure 6:**
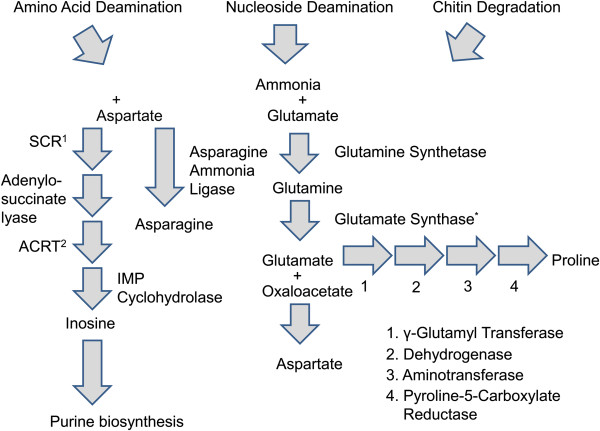
**Proposed mechanisms for direct utilization of ammonia detected in the** ***A. glabripennis*** **midgut.** Several methods for re-incorporating ammonia produced through nucleotide or amino acid deamination reactions into amino acid and nucleosides were reconstructed based on the presence of unigenes in the *A. glabripennis* midgut transcriptome. Ammonia can be directly integrated into glutamine by glutamine synthetase, which can subsequently be converted into glutamate by microbial-derived glutamate synthase detected in the *A. glabripennis* midgut metagenome. Glutamate could subsequently be converted into either proline by a series of reactions catalyzed by γ-glutamyl kinase, dehydrogenase, aminotransferase, and pyrroline-5-carboxylate or aspartate via transaminase, respectively. Furthermore, aspartate can be converted into inosine, which can be directly used for synthesis of purine nucleotides or asparagine via asparagine synthetase. *Microbial in Origin. ^1^Succinylaminoimidazole carboxamide ribotide synthase. ^2^Aminoimidazole carboxamide ribotide transformylase.

The phloem tissue where early instars feed is rich in amino acids relative to the heartwood [77] where older larvae grow and develop. Therefore, recycling waste products of amino acid and nucleotide deamination reactions back into functional amino acids, nucleotides, and other nitrogen-containing compounds may be important to the nitrogen economy in *A. glabripennis* (Figure 6)*.* A number of transcripts with highest scoring BLASTP alignments to enzymes predicted to catalyze deamination reactions and liberating ammonia from a variety of nitrogen-containing compounds were detected in the midgut transcriptome. These included adenine deaminases, cytosine deaminases, nitrilases, amidohydrolases, and chitin-degrading enzymes. Ammonia liberated from nitrogen-containing compounds could be directly converted to glutamine by glutamine synthetase and aspartic acid by aspartate ammonia ligase and indirectly incorporated into the synthesis of purines [78], glutamate, alanine, asparagine, and proline by enzymes encoded by the insect. Furthermore, ammonia or amino acids constructed from recycled ammonia could be shuttled to microbes housed in the midgut to synthesize nonessential or essential amino acids, augmenting or complementing *A. glabripennis’* physiological capabilities. Despite these potential contributions to nitrogen economy, the mechanisms of essential amino acid synthesis and recycling are not clear as the abundance of essential amino acids in woody tissue varies depending on tree species, but are significantly lower than the abundance of nonessential amino acids [16]. As with other insects studied, no pathways for the synthesis and metabolism of essential amino acids (e.g., branched chain and aromatic amino acids) were detected in the midgut transcriptome, although these could be expressed elsewhere. However, full pathways for the synthesis of nine essential amino acids were detected in the *A. glabripennis* midgut metagenome, including histidine, isoleucine, leucine, lysine, methionine, phenylalanine, threonine, tryptophan, and valine, and could serve as key sources of essential amino acids in this insect [25]. Furthermore, the gut community also contained numerous uricase and urease genes, which could be involved in recycling nitrogenous waste products produced by *A. glabripennis* or its gut microbes [25]. Although the waste products from *A. glabripennis* have not been biochemically characterized, all enzymes associated with the urea cycle were detected in the *A. glabripennis* midgut, including several arginase transcripts that catalyze the conversion of arginine to urea, suggesting that urea could be produced in this insect. This urea pathway is also functional in the guts of several other insects [79, 80].

Transcripts predicted to encode several types of digestive proteinases, including serine and cysteine proteinases, were detected, which aid in protein acquisition from glycoproteins cross-linked in the cell wall matrix [17] or from microbes housed in the midgut. By far, serine proteinase unigenes were more expansive than cysteine proteinase unigenes and cystatins (cysteine proteinase inhibitors) were co-expressed and likely repress cysteine proteinase activities [81] in the midgut. These results are consistent with a previous study that reported high serine protease activities and the absence of cysteine and aspartic peptidase activities in the *A. glabripennis* midgut [82]. However, the expression of both cysteine and aspartic proteinases in the midgut suggests that this insect still has the genetic capacity to produce these proteinases under certain conditions and these genes may be maintained in the genome as a strategy to combat digestive proteinase inhibitors produced by host plants. Analysis of transcripts involved in converting compounds in woody tissue into fatty acids and sterols was also conducted (Additional file 1; Supplementary Results).

### Transcripts involved in facilitating interactions with gut microbes

**Figure 7:**
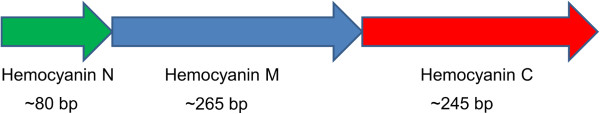
**Structure of hemocyanin enzymes detected in the** ***A. glabripennis*** **midgut transcriptome.** Seven unigenes predicted to encode hemocyanins were detected. Unlike many insect hemocyanins, the hemocyanins produced by *A. glabripennis* had intact and presumably functional copper binding domains. These unigenes were predicted to have involvement in mediating interactions with gut microbes.

Although there is debate about how microbes associated with cerambycid guts contribute to digestive physiology [83], a number of transcripts with putative involvement in mediating interactions with microbes were detected in the *A. glabripennis* midgut transcriptome. Several of these transcripts are likely involved in maintaining host-microbe homeostasis, including transcripts predicted to encode both antifungal and antibacterial proteins, dual oxidases [84], mucin, which forms a protective barrier to protect the midgut from microbial invasion [85], MPA2 allergen proteins, which have antimicrobial properties and are often upregulated during periods of stress [86], and several encapsulation proteins involved in activating innate immune pathways. Seven unigenes predicted to encode hemocyanins were detected (Figure 7). While they primarily function as oxygen carriers in crustaceans, hemocyanins are rare, but not completely absent in insect genomes and their physiological functions are not well characterized [87]. Despite their functional obscurity in insects, they can function as pro-phenol oxidases under certain circumstances, activating innate immune pathways and mediating insect-microbe interactions in the midgut [88]. Hemocyanins have also been hypothesized to serve roles in the degradation of lignin since transcripts encoding hemocyanins are highly expressed in a symbiont-free, wood-feeding marine isopod, but no direct involvement in this process has been demonstrated [89]. Additionally, signal peptides were not observed in any of the hemocyanin unigenes detected in *A. glabripennis* which, if not an assembly artifact, would preclude their involvement in extracellular digestive processes (e.g. lignin degradation).

### Identification of highly expressed genes

Transcripts originating from genomic and mitochondrial ribosomal rRNAs were omitted from this analysis and the unigenes with the top 50 FPKM values (fragments per kilobase of exon per million mapped reads) containing predicted coding regions were identified as highly expressed (Additional file 2). Many of the highly expressed genes identified in the midgut have predicted involvement in stress and immune modulation and included unigenes predicted to encode several MPA2 allergen domain proteins, five carboxylesterases, two cathespins, two encapsulation related proteins, two mucin proteins, a lysosomal lipase, a cytochrome P450, a thaumatin domain protein, and a lectin domain protein. While carboxylesterases and cytochrome P450s have key involvements in detoxification and sterol acquisition, cathespins, encapsulation proteins, mucin proteins, lipases, thaumatin domain proteins and lectins are hypothesized to play fundamental roles in mediating host-microbe interactions. Not surprisingly, GH 48 and GH 5 cellulases and GH 1 β-glucosidases were also highly expressed in the midgut, reflecting the nutritional importance of cellulose to *A. glabripennis*. GH 31 and GH 35 β-galactosidases were also highly expressed, suggesting that galactan polymers present in hardwood hemicellulose or on the cell surface of microbes are also crucial sources of sugar for this insect. Chitin deacetylase unigenes were highly abundant; these enzymes can liberate acetate from insect or fungal chitin, which can be recycled for energy or fatty acid production [90]. Several different types of digestive proteinases were also highly expressed and included M16 peptidases, M14 carboxypeptidases, serine proteinases, and cysteine proteinases, which likely serve key roles in nitrogen extraction from plant or microbial cell wall proteins. In addition to these digestive proteins, several unigenes predicted to encode hypothetical proteins with unknown functions were abundant, suggesting that *A. glabripennis* encodes suites of novel proteins that could be relevant for digestive physiology and development.

### Glycoside hydrolase profile comparisons

**Figure 8:**
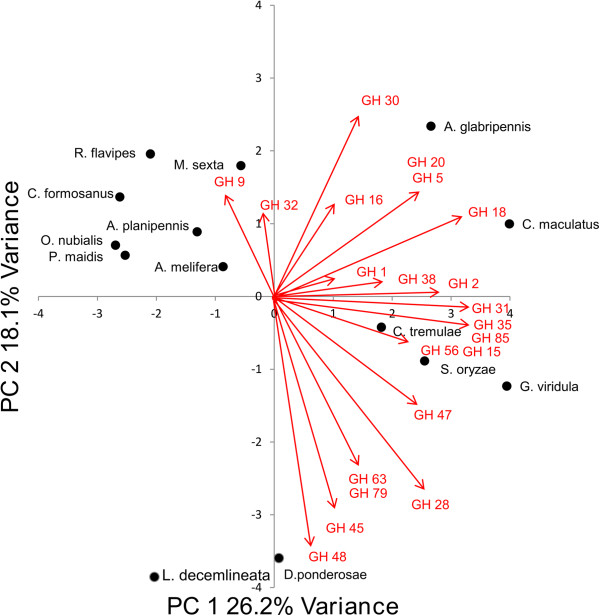
**Multivariate comparison of glycoside hydrolase families detected in the gut transcriptomes of herbivorous insects.** Principal components analysis was conducted to plot the abundance of each glycoside hydrolase family detected in the gut transcriptomes of wood-feeding insects. PCA values were plotted in sample space and variables are displayed as vectors on the PCA biplot. PCA axis 1 and PCA axis 2 explain 26.2% and 18.1% of the variation in the data, respectively. Monte Carlo Permutation Procedure (n = 1000 iterations): p < 0.0001 for PCA 1 and PCA2. No major clustering of insects feeding on similar food sources was detected.

Through comparisons of transcriptome libraries sampled from a variety of herbivorous insects, no major trends were detected with regard to GH profiles and feeding habitats. Euclidean distances between insects that fed on similar substrates were large in many cases and reflected strong differences in GH compositions. Thus, the gut transcriptome libraries did not show any significant clustering trends by food source (Figure 8). For example, *Agrilus planipennis* and *Dendroctonus ponderosae* both feed in phloem and were found in separate planes of the PCA ordination, indicating that there were large differences in GH family composition between these two insects. Likewise, *A. glabripennis*, *Coptotermes formosanus*, and *Reticulitermes flavipes* all feed in wood and were also found in opposite quadrants in the PCA ordination. Although these insects are all capable of producing endogenous cellulases, *A. glabripennis* produces different types of cellulases than the wood- and phloem-feeding insects compared in this study. For example, the two termite species included in this analysis predominately produce GH 9 cellulases, while *A. glabripennis* produces GH 5, GH 45, and GH 48 cellulase transcripts and *Dendroctonous ponderosae* produces GH 45 and GH 48 cellulase transcripts.

**Figure 9:**
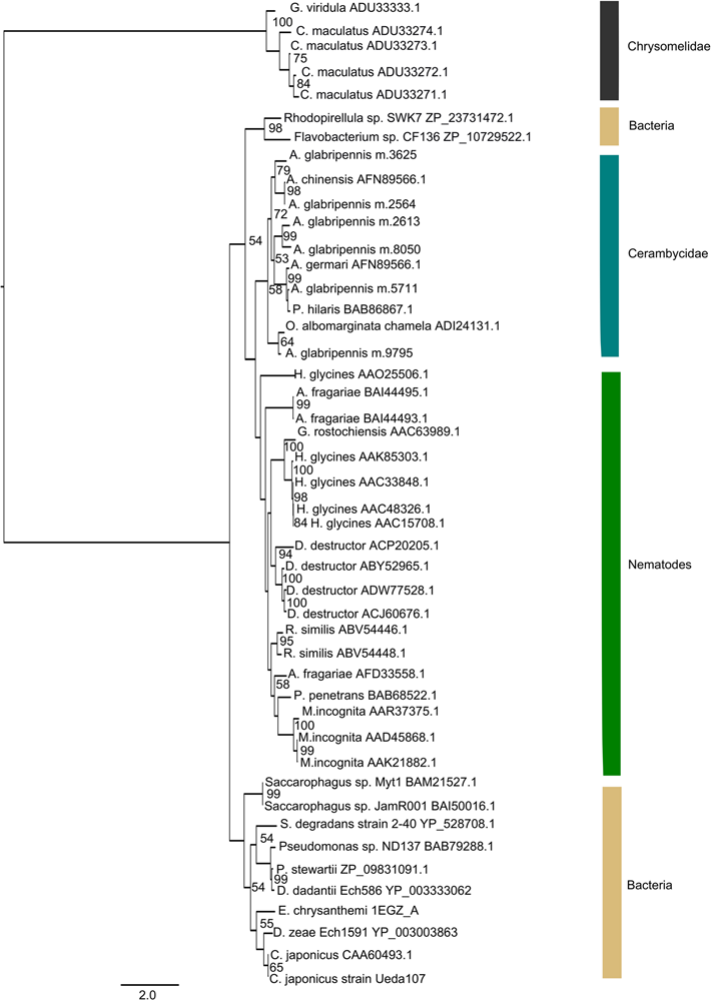
**Phylogenetic analysis of GH 5 cellulases detected in the Coleoptera.** Unrooted maximum likelihood tree for insect, bacterial, and nematode derived GH 5 family proteins was constructed with Garli (version 2.0) using the WAG [91] + I + F + G evolutionary model (n =500 bootstrap replicates). Fully resolved bootstrap consensus trees were compiled using Sum Trees (version 3.3.1) and bootstrap values > 50 are displayed. Nodes are annotated with NCBI protein database accession numbers. Scale bar represents branch lengths.

Despite the lack of clustering by feeding niche, there appeared to be some clustering by phylogenetic relatedness. For example, most cerambycid and chrysomelid beetles were positioned along the positive X axis and, like *A. glabripennis*, some of these insects produce transcripts predicted to encode GH 5, 45, and 48 cellulases, although they feed on very different parts of their host plants. Furthermore, it is interesting to note that GH 5 cellulases have not yet been found in any insect outside the order Coleoptera, but the number of GH 5 cellulases unigenes detected in insect species from this order varied tremendously. While GH 5 transcripts were not detected in association with many coleopterans, the chrysomelids *Gastrophysa viridula* and *Callosobruchus maculatus* encode one and four GH 5 unigenes, respectively. Phylogenetic analysis of translated proteinsequences revealed that, although chyrsomelid GH 5 cellulases and cerambycid (*A. glabripennis*, *Anoplophor achinensis*, *Apriona germari*, *Psacothea hilaris*, and *Oncideres albomarginata chamela*) GH 5 cellulases share a common ancestor, chrysomelid cellulases have rapidly diverged from cerambycid cellulases (Figure 9). In contrast, GH 5 cellulases within the Cerambycidae seem to have multiplied and diversified through gene conversion or gene duplication events and are possibly more adapted to digesting highly insoluble cellulose associated with woody plants.

**Figure 10:**
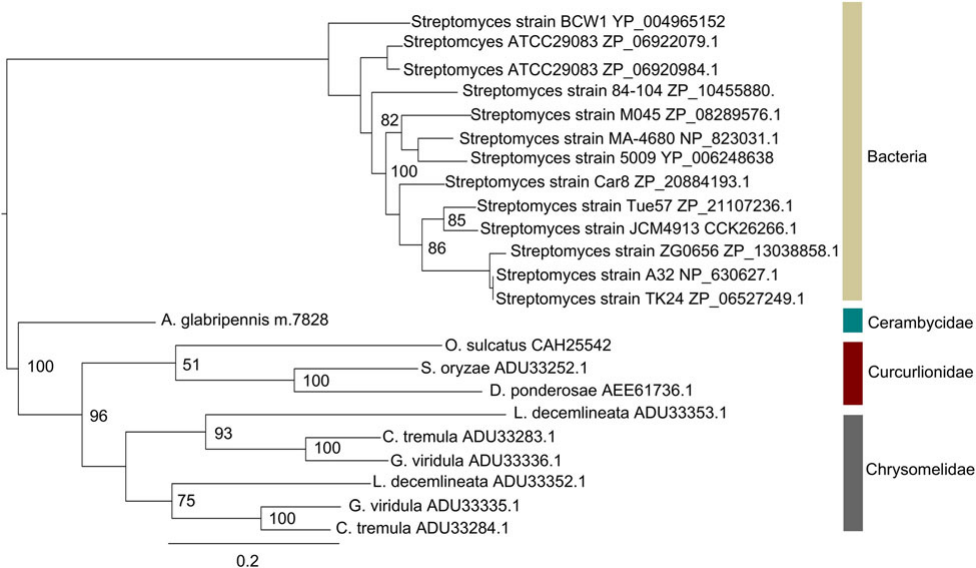
**Phylogenetic analysis of GH 48 cellulases detected in the Coleoptera.** Unrooted maximum likelihood tree for insect and bacterial derived GH 48 family proteins was constructed with Garli (version 2.0) using the LG [92] + I + F + G evolutionary model (n = 500 bootstrap replicates). Fully resolved bootstrap consensus trees were compiled using Sum Trees (version 3.3.1) and bootstrap values > 50 are displayed. Nodes are annotated with NCBI protein database accession numbers. Scale bar represents branch lengths.

**Figure 11:**
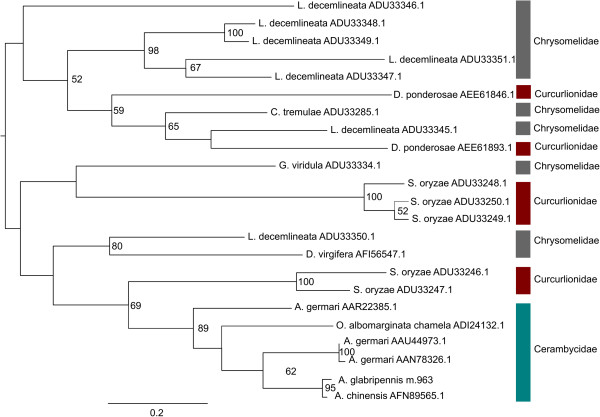
**Phylogenetic analysis of GH 45 cellulases detected in the Coleoptera.** Unrooted maximum likelihood tree for Coleopteran-derived GH 45 family proteins with Garli (version 2.0) using the WAG [91] + G evolutionary model (n = 500 bootstrap replicates). Fully resolved bootstrap consensus trees were compiled using Sum (version 3.3.1) and bootstrap values > 50 are displayed. Nodes are annotated with NCBI protein database accession numbers. Scale bar represents branch lengths.

Furthering the hypothesis that the PCA ordination was primarily driven by phylogenetic relatedness, family-specific trends in abundances of GH families were observed within the Coleoptera. In contrast to GH 5 cellulases, which seem to have multiplied in some cerambycid beetles, GH 45 and GH 48 cellulases were expressed as single copy genes in *A. glabripennis* (Figures 10 and 11). In contrast, members of these GH families have multiplied and diversified in the chrysomelids and curculionid lineages, suggesting that coleopterans have undergone lineage specific adaptations to overcome challenges associated with different feeding regimes. For example, the results of the GH 48 maximum likelihood analysis (Figure 10) suggest that GH 48 enzymes were likely encoded in the genome of the last common ancestor of coleopterans and that they underwent family-specific adaptations. This scenario is supported since GH 48 proteins in each insect associated family formed their own supported clusters in the maximum likelihood tree. In particular, genes encoding GH 48 enzymes were likely duplicated in the Chrysomelidae. All members of this family encode at least two GH 48 proteins and the branching topology suggests that the second GH 48 gene originated directly from the first. Likewise, GH 45 genes have also duplicated and proliferated throughout the chrysomelid and curcurlionid lineages, but the dynamics driving the evolution of this GH family seem to be more complex in comparison to the GH 48 family (Figure 11). In some species, GH 45 genes have rapidly propagated and diversified (e.g. *Leptinotarsa decemlineata*), while in other cases, the insect expressed only a single copy of this gene (e.g. *G. viridula* and *C. tremulae*).

The hypothesis that *A. glabripennis* benefits from microbial enzymes to facilitate nutrient acquisition is supported through the above comparisons. For example, transcripts predicted to encode GH family 18, 20, and 30 chitinases, β-hexosamidases, and glucosylceramidases are strongly associated with *A. glabripennis*. Although these chitin-degrading genes may be important for remodeling the gut peritrophic matrix, which is predominately composed of chitin, they could also play key roles in modulating interactions with fungal taxa associated with the midgut, including yeasts and *Fusarium solani,* a soft rot fungal symbiont of *A. glabripennis*[93, 94]. We hypothesize that the predominance of chitinases allows *A. glabripennis* to derive a portion of its carbohydrate and nitrogen resources from fungal chitinous cell walls, which are composed of polymers of N-acetylglucosamine. Non-entomopathogenic fungi associated with wood-feeding insects have been previously hypothesized to concentrate and/or recycle nitrogen [95] and thus we also hypothesize that the Ascomycota fungal strains found in association with the *A. glabripennis* midgut serve these same purposes.

### Multivariate transcriptome comparisons of gene ontology annotations

**Figure 12:**
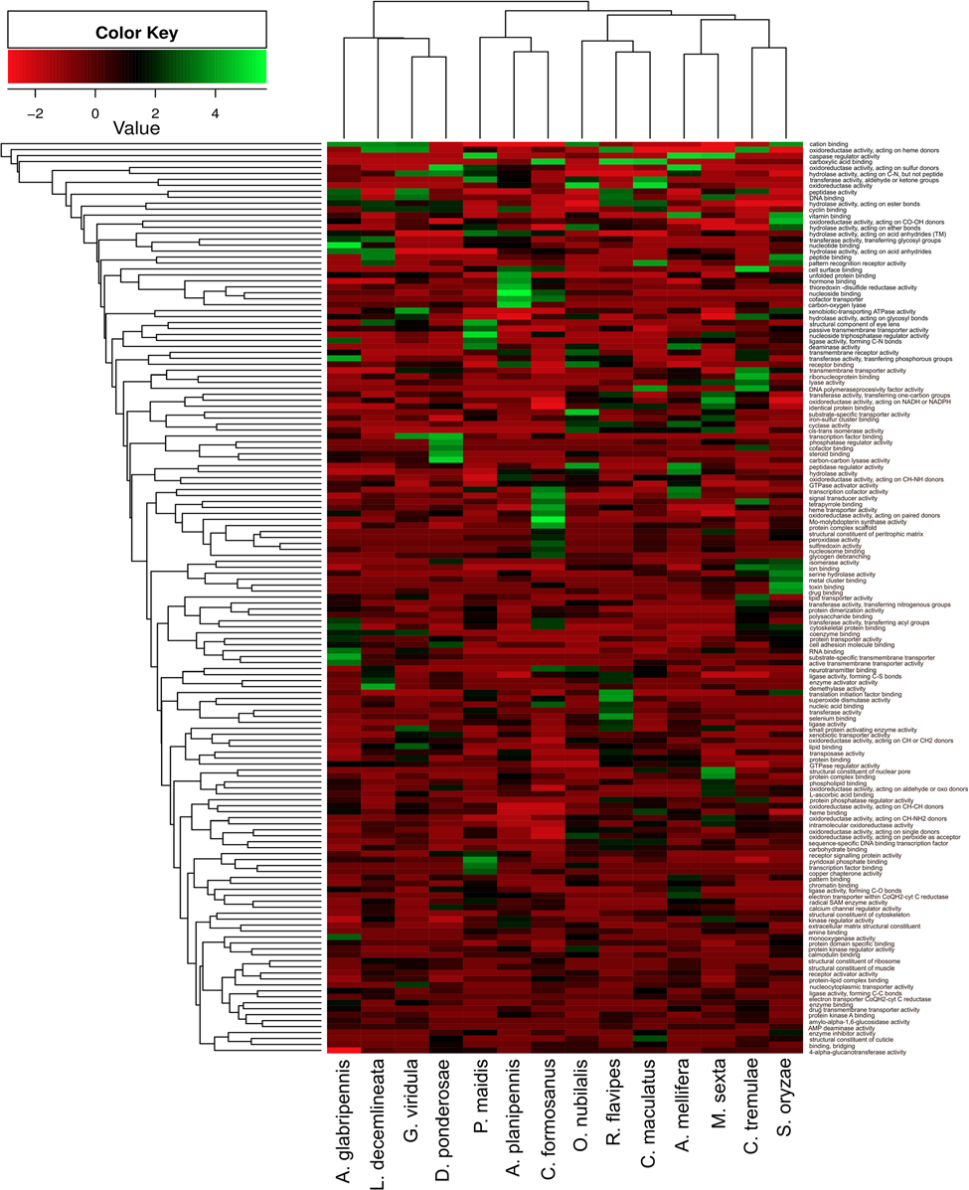
**Two-way cluster analysis of level four gene ontology terms from herbivorous insect gut transcriptomes.** Two-way cluster analysis of level four gene ontology terms was performed to identify potential correlations between abundances of level four GO terms and feeding niche. Clustering did not appear to be driven by food source or phylogenetic relatedness. However, the *A. glabripennis* midgut transcriptome is notably distinct from the other gut transcriptomes included in this comparison.

Like the GH analysis, multivariate comparisons of level four GO categories in midgut transcriptome libraries sampled from a variety of herbivorous insects revealed no significant clustering of transcriptome libraries by feeding habitat. However, phylogenetic relatedness alone did not explain the observed pattern of clustering achieved for the transcriptome comparisons (Figure 12). In addition, subsets of different GO categories were enriched in each transcriptome library included in the comparison, while many of the GO categories were present in approximately the same abundances in each library. Together, these findings suggest that most insects possess similar repertoires of gene families and that these genes have adapted in lineage specific manners optimal for overcoming digestive and nutritional challenges associated with specific feeding habitats and ecological niches. For example, although most insects produce a similar number of GH unigenes (directed at O-glycosyl linkages), the GH family-level comparison uggested that each insect produced its own unique GH profile. Other GO categories that are present in similar abundances in all insects included in this comparison include 4-α-glucanotransferases, heme binding and transporting proteins, and regulatory genes (GTPases, MAP kinases, etc.).

Despite the lack of clustering by food source or phylogenetic relatedness, several trends were detected that distinguish *A. glabripennis* from the rest of the insects included in this comparison that could be pivotal to its ability to digest lignocellulose and other wood polysaccharides and extract nutrients from a broad range of deciduous host trees. For example, in the midgut of *A. glabripennis*, more unigenes and transcript isoforms were produced with predicted monooxygenase and oxidoreductase activities relative to other insects included in this comparison, which could be relevant to its ability to detoxify allelochemicals from its broad range of host plants. Further examination of Pfam domain abundances in each library revealed that unigenes and transcript isoforms predicted to encode carboxylesterases and cytochrome P450s were more abundant in the *A. glabripennis* midgut than many of the other insect libraries sampled. *A. glabripennis* also has the broadest host range of any insect included in this comparison, suggesting that it needs to encode a broader arsenal of detoxification enzymes relative to other insects included in this comparison. Several unigenes predicted to encode digestive peptidases, ligases (forming C-N bonds), and protein transporters were also overrepresented relative to other insect transcriptome libraries, which could be relevant for digesting and assimilating proteins produced by microbes associated with the midgut or from plant cell walls [17]. These digestive peptidases are also overrepresented in *G. viridula*, *C. formosanus*, and *M. sexta* transcriptome libraries. Unigenes associated with hydrolase activity (acting on acid anhydrides) were also highly abundant in *A. glabripennis,* many of which were predicted to encode ATPases and other nucleosidases, DNA binding proteins, RNA binding proteins, nucleotide binding proteins, and transferases involved in transferring phosphorous containing groups. The high abundance of unigenes for these nucleotide binding proteins and nucleosidases is likely associated with the high numbers of unigenes predicted to encode reverse transcriptases, transposases, and integrases that were detected in the midgut. Finally, unigenes predicted to encode proteins with substrate-specific and active transmembrane transporter activities, including major facilitator family (MFS) transporters, were also highly abundant in the *A. glabripennis* midgut. MFS transporters are a diverse group of carriers involved in the absorption of small solutes, including sugar, aromatic amino acids, and other small compounds, which may be involved in assimilation and utilization of small microbial metabolites and/or small metabolites released from the degradation of woody tissue. Taken together, the differences in GH family and level four GO compositions among insects with similar feeding regimes suggest that the ability to degrade polysaccharides found in woody tissue evolved through lineage specific adaptations rather than through convergent evolutionary processes.

### Insights into interactions with microbes

In a recent publication documenting the metabolic potential of the microbiota associated with the *A. glabripennis* midgut, we discovered a taxonomically diverse assemblage of bacteria and fungi primed to make key contributions to digestive physiology, wood digestion, and nutrient acquisition in this system [25]. However, the potential contributions of the beetle to digestive processes were unknown as no comprehensive transcriptomic or genomic resources are currently available for cerambycids; thus, it is difficult to determine precisely how the gut microbes augment or complement physiological processes in the gut. Here, we demonstrate that *A. glabripennis* also has a relatively versatile metabolic potential, producing a number of its own key digestive enzymes. In particular, *A. glabripennis* endogenously produces a diverse suite of detoxification enzymes, including CYP 450s and carboxylesterases, many of which act promiscuously on a diverse array of toxins and are hypothesized to facilitate the degradation and inactivation of host tree defensive compounds. In contrast, comparatively few CYP 450s and carboxylesterases were detected in the microbiome of the beetle gut. Instead, the community predominantly produces enzymes that target specific classes of toxins, including salicylates and other compounds involved in salicylic acid mediated defenses, as well as arsenic, cyanide, and cyanoamino acids. These could enhance endogenous detoxification processes driven by beetle-derived enzymes and could also serve to detoxify many of the defensive chemicals that act directly on the microbial community (e.g. salicylic acid), thereby having indirect deleterious impacts on the beetle. Furthermore, *A. glabripennis* produces several different types of plant cell wall degrading enzymes, including cellulases, β-glucosidases, and xylanases that likely serve a major role in breaking down these prominent wood polysaccharides. However, the community also produces a diverse array of plant cell wall degrading enzymes, which could not only serve to augment the cell wall degrading capacities of *A. glabripennis*, but could also complement these endogenous processes. For example, while the beetle produces enzymes predicted to target β-1, 4 linkages in xylan, several genes predicted to encode enzymes with activity towards ester linkages in xylan were detected in association with the gut community and included acetyl xylan and feruloyl esterases. The presence of these esterases could expedite degradation of xylan in the midgut by cleaving ester linkages that cross-link adjacent hemicellulose chains, exposing sugar residues to beetle-derived hydrolytic enzymes and enhancing digestibility. In addition, although xylose is the second most abundant sugar in deciduous trees, no putative β-xylosidases were detected in the *A. glabripennis* midgut transcriptome. However, gene tags predicted to encode β-xylosidases and enzymes associated with the pentose phosphate pathway were highly abundant in the midgut community, which could possibly serve to convert xylose sugars into compounds that can be directly used by *A. glabripennis* for energy and fatty acid production*.*

In addition, the potential for cooperation between *A. glabripennis* and its gut microbes was also noted with regards to lignin degradation and nitrogen recycling. For example, *A. glabripennis* produces a small pool of transcripts that could facilitate degradation of lignin, including aldo-keto reductases, laccases, and peroxidases. Alone, these enzymes can only facilitate small-scale degradation of the phenolic linkages that comprise a relatively small percentage of the lignin biopolymer. However, larger scale lignin degrading reactions could be accomplished through interactions with enzymes produced by the gut community. For example, the microbial community contained a high abundance of gene tags involved in synthesizing aromatic redox mediators, which could work in tandem with insect-derived laccases to facilitate degradation of the β-aryl ether linkages that predominantly comprise lignin, and genes tags predicted to directly degrade β-aryl ethers and other abundant linkages in lignin.

The beetle requires essential nutrients to complete its development, which are lacking in woody tissue. Complete pathways for the synthesis of all 23 major amino acids and several essential vitamins were detected in the gut community, which could augment the production of non-essential amino acids endogenously synthesized by the beetle and contribute to the production of essential amino acids and other essential nutrients, which the beetle cannot synthesize. Although *A. glabripennis* has abilities to scavenge ammonia, reincorporating it into nonessential amino acids and nucleotides, the community has an expanded capacity to recycle nitrogenous waste products including urea, uric acid, xanthine, and arginine, to potentially reincorporate ammonia into both essential and non-essential amino acids, nucleotides, and other nitrogen-containing compounds. Thus, the community could serve as an additional source of nitrogen and non-esseential amino acids in this high C:N environment. The microbiota also has the capacity to fix atmospheric nitrogen, providing additional sources of nitrogen to both the beetle and members of the gut community.

While this suggests that the beetle collaborates with its gut microbes to facilitate survival in woody tissue, it is unknown which of these microbial pathways are metabolically active in the *A. glabripennis* midgut. Because the transcriptome library was sampled primarily from midgut tissue, few microbial transcripts were detected in this dataset. To gain further insight into transcriptional activity of microbes in the gut, we are currently sequencing RNA collected from the midgut contents to prepare a sample that is more enriched in microbial RNAs. This was the same approach that was used to sequence the metagenome of the *A. glabripennis* midgut and we anticipate that this will expand our abilities to more conclusively model the interactions between *A. glabripennis* and its gut microbes that enhance fitness and/or are required for survival in woody tissue.

## Conclusions

The *A. glabripennis* midgut transcriptome provides the first comprehensive insight into the endogenous digestive capabilities of wood-boring cerambycid larvae as they feed in a highly lignified and nutritionally deficient environment. Comparative transcriptome analysis clearly distinguished the *A. glabripennis* midgut transcriptome from the gut transcriptome libraries of other herbivorous insects that have been previously sampled for sequencing, which may contribute to its long life cycle and ability to feed and develop in a highly lignified food source. Our results highlighted gene categories that were enriched in the *A. glabripennis* midgut transcriptome, which were hypothesized to make key contributions to this insect’s lifestyle, including its ability to colonize a broad range of living host trees. For example, unigenes predicted to encode monooxygenases, carboxylesterases, heat shock proteins, and other detoxification enzymes were highly abundant in the midgut relative to unigenes expressed in the gut transcriptomes of many other herbivorous insects included in this comparison. Furthermore, *A. glabripennis* expressed its own unique profile of GH unigenes for liberating sugars from woody tissue. Our results also highlight deficiencies in endogenous digestive and metabolic pathways that could be supplied by microbes associated with the gut, including enzymes for xylose and pentose sugar utilization, enzymes that facilitate the degradation of lignin, pathways for the synthesis of essential amino acids and nutrients, and pathways for nutrient scavenging. For these reasons, we hypothesize that enzymes derived from midgut-associated microbes complement the expression of insect genes and serve vital roles in the digestive physiology of *A. glabripennis*. This study also substantially expands the genetic resources available for coleopterans by providing transcriptome data for an insect that feeds in the heartwood of healthy host trees. The results presented here may serve as a source for bioprospecting of novel enzymes to enhance industrial biofuels productions and for the pursuit of novel targets for controlling this serious pest.

## Methods

### I. 454 Transcriptome analysis of *A. glabripennis* larvae feeding on a suitable host

Five pairs of adult *A. glabripennis* were allowed to mate and oviposit eggs in potted sugar maple (*Acer saccharum*) trees in a USDA-approved insect quarantine facility at The Pennsylvania State University (University Park, PA). In brief, sugar maple trees were planted in 25-gal nursery containers filled with Fafard 52 pine bark medium (Fafard, Agawam, MA) and were grown at an outdoor nursery until they were 3–4 years old. Several weeks before use in experiments, trees were moved into the quarantine greenhouse to allow for acclimation to greenhouse conditions. Three trees were placed in a walk-in insect cage (~3 m high, 3 m long, and 2 m wide) and five mating pairs of *A. glabripennis* adults were placed in the cage and allowed to mate and lay eggs. After a period of three months, third instar larvae actively feeding in the heartwood of these trees were dissected and midguts were removed and flash frozen in liquid nitrogen. Five midguts were pooled and total RNA was extracted using the RNeasy RNA extraction kit (Qiagen, Gaithersburg, MD) followed by enrichment for mRNA using the PolyA Purist kit (Ambion, Austin, TX). The quality and quantity of the enriched mRNA was assessed using the RNA Nano Assay (Agilent, Santa Clara, CA) and the Nano Drop 1000 spectrophotometer (Thermo-Scientific, Waltham, MA). Approximately 10 μg of enriched RNA were used for double-stranded cDNA library construction using the Stratagene Just cDNA Synthesis kit (Agilent, Santa Rosa, CA). The sequencing library was prepared using 454 GS FLX library adapters (Roche, Branford, CT) and approximately 232,824 shotgun reads (49.1 Mb) were sequenced using 454 FLX chemistry (Roche, Branford, CT). Reads are publically available in NCBI’s Sequence Read Archive (SRA) under accession number [SRX265389] and are associated with Bioproject [PRJNA196436]. Raw reads were trimmed to remove residual sequencing adapters and low quality ends; trimmed reads were quality filtered and assembled using Newbler (Roche, Branford, CT) to produce approximately 2,081 contigs and 1,678 isotigs (e.g. transcripts), while 27,000 singleton reads were not incorporated into the assembly. Short singleton reads were discarded and, to increase the amount of information present in the transcriptome dataset, high quality singleton reads (average quality value >30) exceeding 150 nt in length were concatenated to the assembly and the pooled dataset was utilized in downstream transcriptome comparisons. To reduce noise from sequencing errors or real nucleotide polymorphisms caused by allelic differences from pooling multiple individuals for sequencing, high quality isotigs and singletons were clustered using CD-HIT-EST prior to functional annotation using a sequence similarity threshold of 0.97 to generate a set of unique isotigs and reads, which were analogous to unigenes. These unigenes were screened for noncoding RNAs using tRNAscan (tRNAs) [96] and HMMER [97] (rRNAs) using HMM profiles for archaeal, bacterial, and eukaryotic small subunit (SSU), large subunit (LSU), and 5.8/8 s ribosomal RNAs [98].

The remaining isotigs and reads were annotated by comparisons to the non-redundant protein database using the BLASTX algorithm (BLAST version 2.2.26) [99] with an e-value threshold of 1e^-5^. Microbial- and plant- derived isotigs and singletons were identified using MEGAN (MEtaGenome ANalyzer) [100] based on the least common ancestor of the top five highest-scoring BLASTX alignments and were removed from the dataset since this study focused solely on the beetle’s contribution to wood digestion. Unigenes were assigned to Gene Ontology terms using Blast2GO [101] while unigenes involved in carbohydrate metabolism were detected and classified into glycoside hydrolase (GH) families using HmmSearch [102] to scan for Pfam A derived HMMs [103]. Gene ontology assignments and GH and Pfam annotations were used in downstream comparisons to gut derived transcriptome libraries from other herbivorous insects.

### Comparison to other insect Gut transcriptome libraries to identify groups of ESTs associated with feeding in wood

EST and transcriptome libraries from other plant- and wood-feeding insects were analyzed for similarities and differences to the *A. glabripennis* midgut transcriptome library in an attempt to identify groups of insect-derived transcripts encoding digestive enzymes that were associated with feeding in wood. Publically available insect gut transcriptomes from insects feeding on plant materials, including wood, phloem, leaves, stored plant materials (starches), and pollen housed in NCBI’s SRA (454 pyrosequencing) or EST database (Sanger sequencing) were downloaded (Table 6). Midgut 454 libraries currently available in the Sequence Read Archive (SRA) include honey bee (*Apis mellifera*), emerald ash borer (*Agrilus planipennis*) [104], green dock beetle (*Gastrophysa viridula*) [42], poplar leaf beetle (*Chrysomela tremulae*) [28], rice weevil (*Sitophilus oryzae*), Colorado potato beetle (*L. decemlineata*), and tobacco hornworm (*Manduca sexta*) [105]. Sanger-derived EST libraries available in the EST database include corn plant hopper (*Peregrinus maidis*) [106], European cornborer (*Ostrinia nubilalis*) [107], mountain pine beetle (*Dendroctonus ponderosae*) [108], and termites (*Coptotermes formosanus* and *Reticulitermes flavipes*) [109, 110].

**Table 6:** **454 and Sanger EST gut transcriptome libraries from herbivorous insects**

Order	Family	Genus	Food	Number reads	NCBI Accession
Blattodea	Rhinotermidae	*Coptotermes*	Wood	142,738	345139168-345281906
		*Reticulitermes*	Wood	63,680	197217790-197281650
Coleoptera	Buprestidae	*Agrilus*	Phloem	126,185	SRX018276
	Cerambycidae	*Anoplophora*	Wood	277,000	N/A
	Chrysomelidae	*Callosobruchus*	Stored product	909,444	SRR037004
		*Chrysomela*	Leaf	277,780	SRR037007
		*Gastrophysa*	Grasses	1,234,472	SRR037001
		*Leptinotarsa*	Leaf	839,061	SRR037005
	Curculionidae	*Dendronctonous*	Phloem	24,880	254003271-254028151
		*Sitophilus*	Stored product	926,752	SRR037006
Diptera	Cecidomyiidae	*Mayetolia*	Sap	118,107	259155064-259273171
Hemiptera	Delphacidae	*Peregrinus*	Sap	120,595	282603884-282720983
Hymenoptera	Apidae	*Apis*	Pollen	622,279	SRX025528
Lepidoptera	Crambidae	*Ostrinia*	Leaf	24,103	254003208-254027311
	Sphingidae	*Manduca*	Leaf	432,961	SRR017588

The libraries were assembled and annotated using the same annotation procedure described for the *A. glabripennis* 454-based assembly with a particular emphasis on protein family (Pfam) domains, [103], Gene Ontology terms, and carbohydrase enzyme (cazyme) family classifications [36], which were utilized in comparisons to the *A. glabripennis* 454-based assembly. Microbial- and plant- derived isotigs and singletons in all assemblies were identified using MEGAN (MEtaGenome ANalyzer) [100] and were removed from the datasets prior to comparisons. Due to differences in sequencing, depths, normalization,and library preparation procedures, assembly metrics varied among libraries (Table 7). As this may introduce sampling biases in downstream comparisons, contigs and high quality reads were normalized *in silico* using CD-HIT-EST [111] to remove redundant reads and contigs to generate a set of unigenes. Prior to performing multivariate comparisons, the length distributions for transcripts and singletons with GO annotations were plotted for each library to ensure the assemblies of the libraries were similar and that major library biases were not responsible for driving the similarities and differences observed in the multivariate comparisons (Additional file 1: Figure S2).

**Table 7:** **Transcriptome assembly and annotation metrics from herbivorous insects included in glycoside hydrolase and Pfam comparisons**

Genus	Number of Unique Reads and Isotigs	Number of Insect Reads and Isotigs	Number of Reads and Isotigs classified as ncRNA	Number of Reads and Isotigs with Gene Ontology Assignments^*^	Number of Reads and Isotigs with KEGG Assignments^*^	Number of Reads and Isotigs with Pfam Domains^*^
*Coptotermes*	33,662	5,728	91	2,187	592	2691
*Reticulitermes*	8,616	2,112	312	1,141	352	1,665
*Agrilus*	29,773	8,614	1,119	4,069	1,230	4,715
*Anoplophora*	20,587	9,109	78	4,331	1,630	4,591
*Chrysomela*	48,675	14,606	48	6,004	1,925	7,888
*Gastrophysa*	45,675	14,783	66	5,720	1,711	10,532
*Leptinotarsa*	81,335	20,037	57	4,385	1,669	5,764
*Callosobruchus*	68,336	20,880	84	7,047	2,045	13,703
*Dendroctonous*	4,328	2,876	130	1,499	423	2,240
*Sitophilus*	56,592	17,846	96	6,278	1,869	11,796
*Mayetolia*	3,895	2,225	536	1,409	441	1,834
*Peregrinus*	19,297	8,740	55	3,957	1,014	6,483
*Apis*	42,370	17,008	179	5,602	1,882	7,110
*Ostrinia*	2,713	1,670	84	2,394	219	1,419
*Manduca*	82,488	13,638	179	6,210	1,957	7,439

*Only insect-derived reads used for these annotations.

### Multivariate transcriptome library comparisons

GH family assignments detected in the *A. glabripennis* 454-based transcriptome assembly were compared to GH family assignments from transcriptomes and EST libraries sampled from herbivorous insect guts feeding on a diversity of plants that varied in carbohydrate composition. This was done to identify potential correlations between carbohydrases associated with insects that feed in similar niches. Data were normalized by the total number of GH domains detected in each library and a compositional dissimilarity matrix was constructed based on Euclidean distance. The standardized data were further analyzed using unconstrained Principal Components Analysis to plot samples in multidimensional space using the R statistical package with the ‘vegan’ library. PCA ordination was selected because the data were determined to be linear by detrended correspondence analysis (DCA) (Beta diversity <4).

To identify functional similarities between insects with similar feeding habitats, a multivariate comparison of level four Gene Ontology (GO) terms identified in the gut transcriptomes of herbivorous insects was performed. To reduce sampling bias due to differences in library sizes and assembly metrics, a custom python script was used to subsample (with replacement) level four GO assignments from 675 reads and isotigs from each library. Data were log transformed, centered, and a compositional dissimilarity matrix of transcriptome libraries was constructed based on Spearman correlation coefficients. Two-way clusters were generated with Ward’s method using the R statistical package and the ‘vegan,’ ‘cluster’, ‘gplots,’ and ‘Biobase’ libraries.

### Phylogenetic analysis

Multiple amino acid sequence alignments were generated using ClustalW [112] and alignments were manually trimmed and edited using MEGA 5 [113]. ProTest [114] was used to predict optimal evolutionary models for maximum likelihood analysis using Akaike Information Criteria (AIC) [115]. Unrooted phylogenetic trees were constructed using Garli (version 2.0) [116]; evolution was simulated for 500,000 generations or until likelihood scores reached convergence and non-parametric bootstrap analysis was conducted to generate support for branching topology (n = 500 bootstrap pseudoreplicates). Fully resolved bootstrap consensus trees were compiled using Sum Trees version 3.3.1 [117] and branch lengths less than 1e^-8^ were collapsed.

### II. Identification of highly expressed genes in the *A. glabripennis* midgut

To generate more full length transcripts, enhance transcript discovery, and identify highly expressed genes in the *A. glabripennis* midgut, short paired-end reads were incorporated into the assembly. Third instar midguts were dissected and total RNA extracted as described above. Insect-derived ribosomal RNA was depleted from the sample using MicrobEnrich (Ambion, Austin, TX), replacing the MicrobEnrich capture oligo mix with custom oligos that were complementary to insect 18 s and 28 s rRNAs (oligo sequences obtained from Ambion, Austin, TX; oligos purchased from Integrated DNA Technologies, Coralville, IA) (Additional file 3: Table S1 and Additional file 1: Supplemental Methods), while MicrobExpress (Ambion, Austin, TX), was used to deplete the sample of bacterial derived 16 s and 23 s rRNAs. The quality and quantity of the enriched mRNA was assessed using the RNA Nano Assay (Agilent, Santa Clara, CA) and the Nano Drop 1000 spectrophotometer (Thermo-Scientific, Waltham, MA). The library was prepared using TruSeq RNA Library Prep Kit (Illumina, San Diego, CA), omitting the polyA enrichment step, and the library was enriched for 175 nt fragments so that paired end reads overlapped by 30 nt. 130 million 100 bp read pairs (36 Gb) were generated using the Illumina HiSeq 2000 platform. To improve overall transcriptome assembly metrics and ultimately improve the ability to detect and annotate expressed genes, 454 and Illumina reads were co-assembled with Trinity. In brief, 10 million 101 × 101 Illumina paired end reads (175 nt fragments) were simulated from 454 isotigs and singletons generated by Newbler using wgsim [118]. To reduce the coverage of highly expressed genes and improve the ability to assemble unigenes and transcript isoforms originating from lowly expressed genes, k-mers (k = 25) from Illumina and simulated PE reads were normalized to 30X coverage using digital normalization. Normalized reads were assembled with Trinity (version r2012-10-05) [119] and TransDecoder was used to predict putative protein coding regions using Markov models trained using the top 500 longest ORFs detected in the *A. glabripennis* transcriptome dataset. Coding regions were annotated through comparisons to the non-redundant protein database using BLASTP with an e-value threshold of 1e^-5^. Unigenes with BLASTP alignments were classified into Gene Ontology (GO) and KEGG terms using Blast2GO [101] and HmmSearch [102] was utilized to search for Pfam A derived HMMs [103], which were used for functional annotations and GH family assignments. Unigenes were also assigned to KOG categories (clusters of orthologous genes for eukaryotes) using RPS-BLAST [120]. Illumina reads were mapped to the hybrid assembly using Bowtie [121], expression levels were calculated using RSEM [122], and FPKM (fragments per kilobase of exon per million mapped reads) values were used to normalize read counts [123]. Unigenes and transcript isoforms with less than five mapped reads were flagged as spurious and were removed from the final assembly. Since co-assembly should improve the ability to assemble full-length transcripts, SignalP was used to detect unigenes and transcript isoforms with discernible signal peptides [124] that could encode digestive proteins secreted into the midgut lumen. Raw Illumina reads are available in the NCBI SRA database under the accession number [SRX265394] and associated with Bioproject PRJNA196436. Assembled insect-derived transcripts containing predicted coding regions generated from co-assembly of 454 and Illumina paired end reads are publically available in NCBI’s Transcript Shotgun Assembly database under the accession number [GALX00000000].

### Availability of supporting data

Raw 454 reads are available in the NCBI SRA database under accession number [SRX265389]. Raw Illumina reads are available in the NCBI SRA database under the accession number [SRX265394] and associated with Bioproject PRJNA196436. Assembled insect-derived transcripts containing predicted coding regions generated from co-assembly of 454 and Illumina paired end reads are publically available in NCBI’s Transcript Shotgun Assembly database under the accession number [GALX00000000]. Alignments and phylogenetic trees used in this study were deposited in TreeBase: http://treebase.org/treebase-web/search/study/summary.html?id=14849.

## Electronic supplementary material

Additional file 1: **Supplemental Methods, Supplemental Results, Figure S1, and Figure S2. Figure S1.** KOG assignments for transcripts with predicted signal peptides. **Figure S2.** Length distributions of annotated transcripts and singletons used in transcriptome comparisons. (DOCX 422 KB)

Additional file 2: **FPKM (Fragments Per Kilobase of transcript per Million mapped reads) values for assembled transcripts.** (XLSX 10 KB)

Additional file 3: **Oligo sequences used to deplete RNA libraries of insect-derived rRNAs**. (XLSX 2 MB)
