# Supplementary material for: Midgut transcriptome profiling of Anoplophora glabripennis, a lignocellulose degrading cerambycid beetle
Source: BMC Genomics. 2013 Dec 4;14(1):850. doi: 10.1186/1471-2164-14-850 (PMC4046674; doi:10.1186/1471-2164-14-850)
Supplement: Supplementary file 1 — Additional file 1: Supplemental Methods, Supplemental Results, Figure S1, and Figure S2. Figure S1. KOG assignments for transcripts with predicted signal peptides. Figure S2. Length distributions of annotated transcripts and singletons used in transcriptome comparisons. (DOCX 422 KB) [file 12864_2013_5589_MOESM1_ESM.docx]

**Supplemental Methods:**

**Preparation of Custom Oligo Mixture for MicrobEnrich:**

100 uM stock solutions for each oligo were purchased from IDT (Coralville, IA). 6 µL (0.6 nmol) of 18S-565, 18S-1270, 18S-1356, 28S-1341, 28S-2438, 28S-3258, 28S-3258, and 28S-3365 and 10 µL 28S-349 (1.0 nmol) were added to 148 µL RNase-free TE buffer (1.5 M, pH=8.0) bringing the final concentration of 18S-565, 18S-1270, 18S-1356, 28S-1341, 28S-2438, 28S-3258, 28S-3258, and 28S-3365 to 2.9 µM each and 28S-349 and to 4.9 µM. Insect rRNAs were subtracted from the total RNA extraction using MicrobEnrich following the manufacturer’s protocol, but replacing the 4 uL capture oligos supplied with the kit with 4 uL of the custom oligo mixture.

**Supplemental Results:**

**Analysis of Transcripts Predicted to Encode Secreted Proteins:**

Approximately 700 transcripts with predicted signal peptides for extracellular targeting were detected in the assembly and could serve key roles in the large scale degradation of woody tissue and nutrient extraction (SFigure 1). Not surprisingly, the many of these transcripts had predicted involvement in carbohydrate and amino acid transport and metabolism, but a function for many transcripts could be not be directly inferred since many transcripts were classified to ‘general functional prediction’ and ‘function unknown’ KOG categories. Although the majority of proteins assigned to the KOG category ‘function unknown’ had highest scoring BLAST alignments to uncharacterized and hypothetical proteins, a subset of the proteins assigned to the ‘general functional prediction’ category could be conclusively characterized and included several carboxylesterases, chitinases, α/β hydrolase proteins, peroxidases, surface mucin proteins, and cysteine proteinases. Further, several transcripts with predicted involvement in defense were also detected, including several transcripts predicted to encode thioredoxins, which likely act as antioxidants and destroy reactive oxygen species [[142](#_ENREF_142)] that may be generated during large scale lignin metabolism that occurs in the gut, and UDP-glucuronsyl transferases, which are involved in conjugative inactivation and subsequent elimination of exogenous xenobiotic compounds.

Not surprisingly, many of the transcripts predicted to encode cell wall degrading enzymes also had predicted signal peptides, including many of the α-mannosidases, β-galactosidases, β-glucosidases, endo-β-1,4-glucanases, β-glucuronidases, and β-fructofuranoisdases. Interestingly, only a subset of the xylanase isoforms had predicted signal peptides, suggesting that only some of these transcripts encode secreted enzymes and that some of the isoforms might have different intracellular roles in processing smaller oligosaccharides (e.g. xylobiose) released from large scale hemicellulose digestion or could serve other digestive functions. Chitinases, trehalases, and β-hexosamidases are also highly represented, which could be involved in remodeling the peritrophic matrix or could be involved in digesting the chitinous cell walls of fungi found in association with the gut. Furthermore, several transcripts predicted to encode enzymes involved in metabolizing proteins and lipids also had predicted signal peptides and could be relevant for digesting proteins embedded in the plant cell wall matrix or lipids produced by gut microbes. Many of the cysteine and serine proteinases with putative involvement in nitrogen acquisition had predicted signal peptides, including several carbon-nitrogen hydrolase proteins, which are likely involved in catalyzing amino acid or nucleotide deaminase reactions for nitrogen recycling processes. In terms of lipid metabolism, several transcripts predicted to encode lysosomal acid phosphatases and palmitoyl protein thioesterases were detected and could be involved in digesting ester-linked fatty acyl groups from microbial cell wall proteins during lysosomal-mediated degradation.

**Sterol synthesis:**

Although the sterol synthesis pathway is blocked at several steps in insects, *A. glabripennis* and most other insects still maintain partial cholesterol synthesis pathways, many of which are still transcriptionally and translationally active. Because these pathways are blocked, insects usually have to acquire cholesterol from their diet or rely on symbiotic microbes for their production. Several transcripts predicted to encode melvonate pathway enzymes were detected in the midgut, including hydroxymethylglutaryl-coA reductase, which catalyzes the first step in the pathway and converts (R)-melvonate to (S)-3-hydroxy-3-methylglutaryl-CoA. Other transcripts predicted to encode enzymes from this pathway included transcripts annotated as farnesyl pyrophosphatase, isopentenyl-diphosphate delta-isomerase (IPP:DMAPP), 24-dehydrocholesterol reductases, 3-hydroxy-3-methylglutaryl coenzyme A synthase, and estradiol 17 beta-dehydrogenase. In addition, despite their prolific roles in detoxification pathways, cytochrome P450s and carboxylesterases could potentially participate in cholesterol synthesis or the conversion of plant phytosterols or microbial-derived ergosterols into sterols and, eventually, into pheromones. Additionally, many transcripts with sterol-sensing or oxysterol binding domains were also detected and may play key roles in metabolizing pheromones or internalizing ergosterols or phytosterols acquired from fungal symbionts or plants, respectively.

**Fatty Acid Synthesis:**

Although fatty acids, sterol esters, and waxes can be found in the sapwood of deciduous host trees where *A. glabripennis* larvae initially feed after hatching, they are present in very low levels in the heartwood were older larvae grow and develop {Leone, 1998 #2600} . Thus, older larvae must have mechanisms in place to conserve or recycle fatty acids or synthesize them from compounds present in the heartwood. We have previously shown that yeasts associated with the gut possess the machinery to convert xylose and other sugars to acetate, which can be used as building blocks for fatty acid production by *A. glabripennis* (Scully et al, submitted). *A. glabripennis* is capable of converting acetate and pyruvate produced by gut microbes (or through glycolysis) to acetyl coA through acetyl-CoA carboxylases and pyruvate decarboxylases respectively.

However, an alternate mechanism for converting components of chloroplasts into medium-chain fatty acids was detected in the *A. glabripennis* midgut transcriptome, which may allow the insect to synthesize fatty acids without the aid of gut microbes. Several transcripts predicted to encode 2-hydroxyphytanoyl-coA ligase were detected in the midgut, which can be intricately involved releasing phytol (a component of chlorophyll) through the fermentation of plant materials in many rumen-associated bacteria. Through a series of reactions involving α-oxidation and peroxisomal enzymes, phytol is converted to phytanoic acid and eventually into pristanic acid, which can be incorporated into medium chain fatty acids. Although the cells found in heartwood are typically empty and dead, cells found in the sapwood and cambium contain a mixture of live and dead cells [[152](#_ENREF_152)] and may still contain remnants of chloroplasts that could be used as a source of nutrients by *A. glabripennis*. Full suites of transcripts predicted to encode enzymes involved in fatty acid synthesis and storage were detected in the midgut transcriptome, including fatty acid synthases, fatty acid elongases, phospholipid synthesis proteins, fatty acid desaturases and triglyceride synthesis proteins (Figure Fatty Acid).

**Supplemental Figures:**


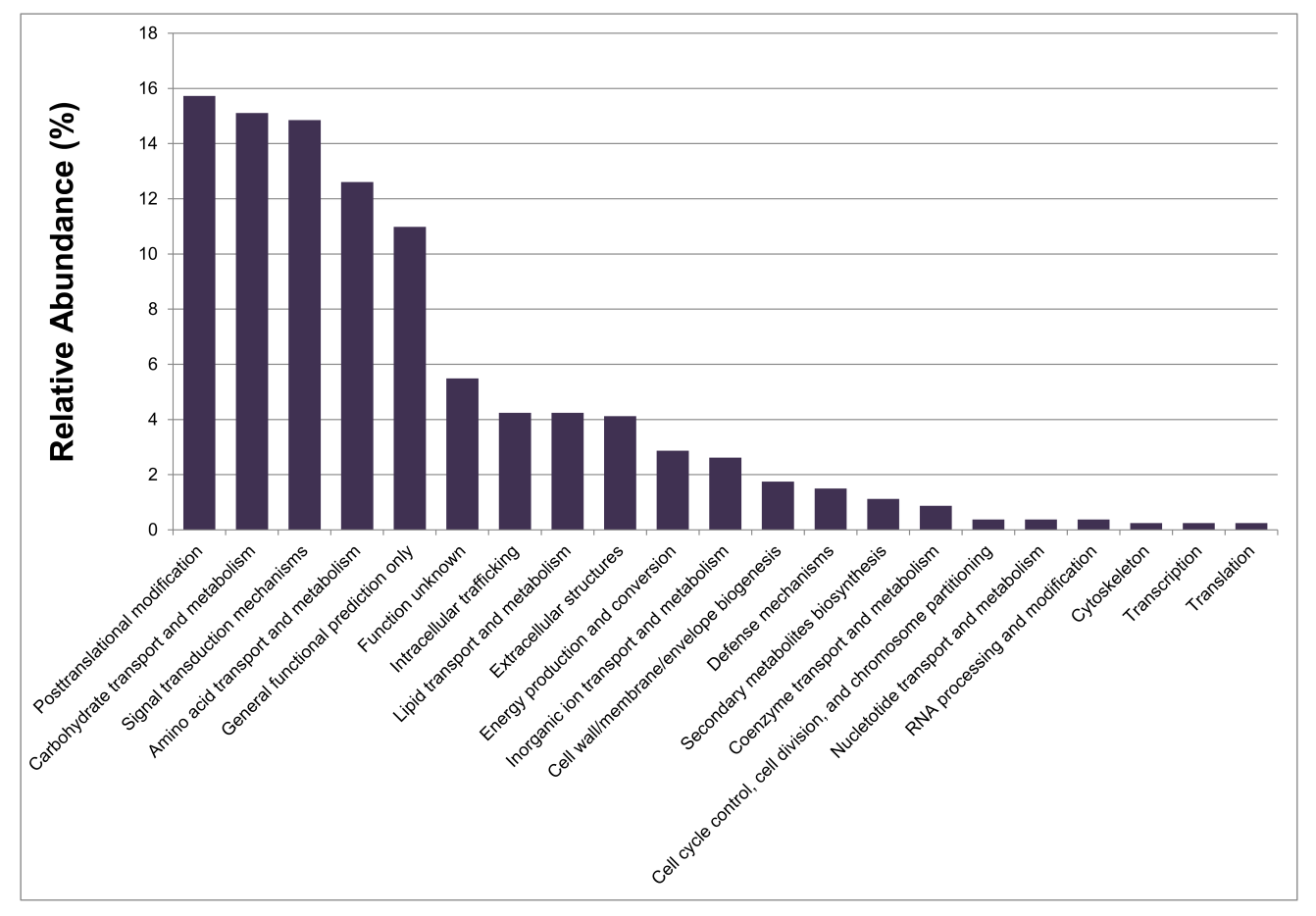


Figure S1. KOG assignments for transcripts with predicted signal peptides.


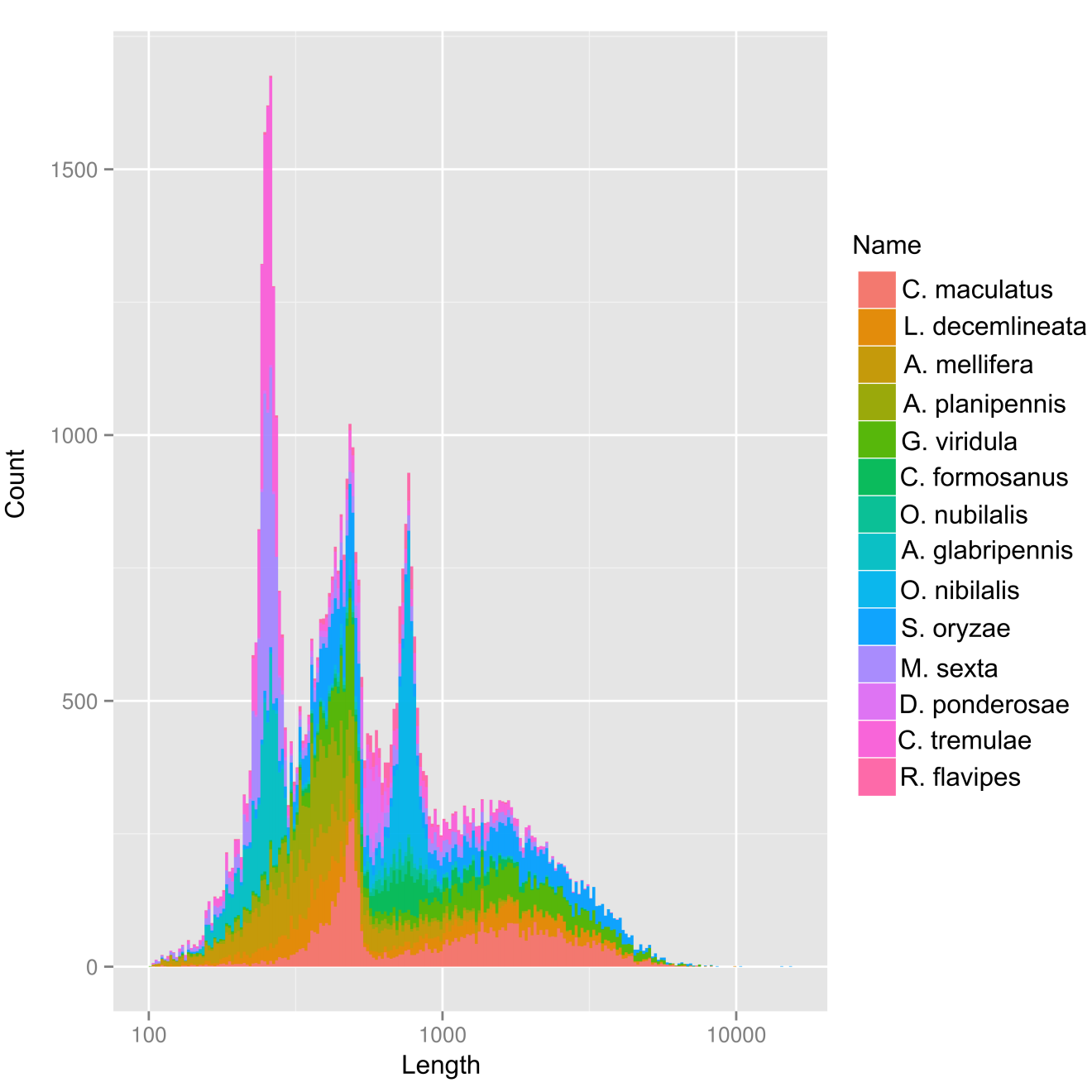


Figure S2. Length distributions of annotated transcripts and singletons used in transcriptome comparisons.
